# Preclinical Evidence and Mechanisms of Traditional Chinese Medicine Polysaccharides Regulating the Gut–Lung Axis via Gut Microbiota in Respiratory Diseases

**DOI:** 10.3390/nu18183087

**Published:** 2026-09-21

**Authors:** Xin-Qian Rong, Xiao-Meng Zhang, Cheng Lu, Yong Tan

**Affiliations:** Institute of Basic Research in Clinical Medicine, China Academy of Chinese Medical Sciences, Beijing 100700, China; rongxinqian2016@163.com

**Keywords:** traditional Chinese medicine polysaccharides, gut–lung axis, gut microbiota, polysaccharide utilization loci, pharmacological mechanisms

## Abstract

Traditional Chinese medicine (TCM) polysaccharides are important active components of traditional Chinese medicine, and their anti-inflammatory, immunomodulatory, and mucosal barrier protective effects have attracted widespread attention. Since most traditional Chinese medicine polysaccharides are difficult to be completely digested and absorbed in the gastrointestinal tract, their interventional effects on respiratory diseases may be closely related to the selective utilization by gut microorganisms and the regulation of the gut–lung axis. Therefore, the structural and functional interactions between traditional Chinese medicine polysaccharides and gut microbiota have gradually become an important direction in traditional Chinese medicine pharmacology and microecology research. This review mainly explores two core questions: first, how traditional Chinese medicine polysaccharides that can act on the gut–lung axis drive candidate gut bacteria and metabolic networks; second, how polysaccharide-driven candidate bacteria and metabolic networks regulate pulmonary infection, allergic inflammation, chronic airway injury, and fibrosis through the intestinal mucosal barrier, immune cell reprogramming, and cross-organ signal transmission. Accordingly, this narrative mechanistic review examines selected preclinical studies on traditional Chinese medicine polysaccharides regulating the gut–lung axis, focusing on summarizing the continuous mechanisms by which different polysaccharides promote the proliferation of related microbial communities and metabolite production through selective feeding of gut microorganisms, stabilize intestinal mucosal immunity, and exert pulmonary interventional effects. This review aims to deepen the understanding of the interaction mechanisms among traditional Chinese medicine polysaccharides, microorganisms, and the host, provide some reference for establishing a mechanistic evidence framework for gut–lung axis research and screening potential active polysaccharides with relevant structures and microbiota effects.

## 1. Introduction

Decoction is the main form of clinical application of traditional Chinese medicine, and traditional Chinese medicine polysaccharides are a class of macromolecular components with relatively high content in decoctions. In the past, because traditional Chinese medicine polysaccharides are difficult to be completely absorbed by the body, they were often removed as inactive components during processes such as “alcohol precipitation for impurity removal.” With the development of polysaccharide chemistry and microecological pharmacology, their immunomodulatory, anti-inflammatory, and mucosal protective effects have gradually attracted attention [1]. Previous studies have shown that after oral administration, traditional Chinese medicine polysaccharides can exert effects through intestinal mucosal absorption, microfold (M) cell uptake, and degradation and transformation by gut microorganisms, while for polysaccharides with larger molecular weights and complex structures, degradation and metabolism involving gut microorganisms are particularly important [2]. This is mainly attributed to the limited types of glycoside hydrolases encoded by the human body itself, which make it difficult to fully degrade various plant- and fungus-derived non-starch polysaccharides [3]. Therefore, a considerable portion of traditional Chinese medicine polysaccharides can reach the distal intestine and subsequently become carbon sources available to gut microorganisms. In recent years, various traditional Chinese medicine polysaccharides have shown certain protective effects in models of viral pneumonia, bacterial lung injury, allergic airway inflammation, chronic airway injury, and pulmonary fibrosis, and their pharmacological mechanisms are mostly explained by changes in Toll-like receptor 4 (TLR4)/nuclear factor kappa B (NF-κB), NLR family pyrin domain containing 3 (NLRP3), mitogen-activated protein kinase (MAPK), and other pathways in lung tissue [4]. However, most studies have used oral administration, and the exposure of the parent forms of polysaccharides in lung tissue is usually low [5]. Changes in pulmonary signaling pathways can explain the terminal link of the pharmacological effect, but cannot fully explain its initial process. For example, related studies on *Houttuynia cordata* polysaccharides have shown that they did not exhibit direct anti-influenza virus activity in vitro, whereas oral administration could alleviate lung injury in influenza A (H1N1)-infected mice, and intravenous administration did not produce the same effect. Meanwhile, their regulation of the T helper 17 (Th17)/regulatory T (Treg) cell balance first appeared in gut-associated lymphoid tissue and was subsequently observed in lung tissue [6,7]. This suggests that the intestine is not only the site of absorption and metabolism of traditional Chinese medicine polysaccharides, but may also be an important starting point for their distal pulmonary effects.

After entering the intestine, traditional Chinese medicine polysaccharides are not utilized indiscriminately by all bacteria. The monosaccharide composition, glycosidic bond types, arrangement of main chains and branches, degree of branching, molecular weight, and spatial conformation of polysaccharides jointly determine their substrate characteristics; modifications such as acetylation, methylation, and sulfation further alter their solubility, charge properties, and enzymatic degradation difficulty. Correspondingly, gut bacteria need to possess matching carbohydrate-active enzymes (CAZymes) and polysaccharide utilization loci (PUL) to complete the recognition, binding, cleavage, and transport of specific polysaccharides [8]. Our previous studies have also shown that the interaction between traditional Chinese medicine polysaccharides and gut microorganisms is essentially a process of mutual matching between polysaccharide structures and microbial enzyme systems [2]; on this basis, traditional Chinese medicine polysaccharides are not fermentation substrates in the general sense, but rather microbiota-regulating substrates with structurally encoded characteristics [9]. Recent studies have further supported this structural selectivity. A study involving 20 medicinal polysaccharides and 28 human gut *Bacteroides* and *Parabacteroides* species found that different medicinal polysaccharides had clearly different species- and strain-level utilization profiles. For example, Dendrobium glucomannan could be utilized by *Bacteroides uniformis* carrying specific PUL, in which glycoside hydrolase family 26 (GH26) is the key enzyme for its degradation of plant mannans [10]. This study also showed that after primary degraders perform extracellular or periplasmic degradation of polysaccharides, the released oligosaccharides, monosaccharides, and intermediate products such as acetate, lactate, and succinate can be further utilized by other bacteria, thereby forming a cross-feeding network [10]. The direct result of this cross-feeding network is that a single polysaccharide may first enrich a small number of bacteria capable of initiating degradation, and then influence butyrate-producing bacteria, propionate-producing bacteria, and other microbial community members through metabolite exchange, thereby driving the microbial metabolic network.

The polysaccharide-driven microbiota and metabolic network provide a material basis for regulating the gut–lung axis. The gut–lung axis is a bidirectional connection between the intestine and the lung maintained jointly by microbial metabolism, mucosal immunity, and circulatory transport [11,12]. Signal transmission from the intestine to the lung generally includes three types of pathways: first, short-chain fatty acids, tryptophan derivatives, aromatic metabolites, and secondary bile acids enter the portal vein and systemic circulation and act directly on lung epithelial cells and intrapulmonary immune cells [13]; second, the local intestinal metabolic environment alters the differentiation and activation states of dendritic cells, macrophages, and T cells, which subsequently affect the lung through cell migration or circulating immune mediators [14]; third, intestinal barrier damage allows lipopolysaccharide, peptidoglycan, and other microbial-associated molecules to enter the circulation, leading to abnormal preactivation of alveolar macrophages and lung epithelial cells [15]. Existing studies have shown that fermentable fiber and the propionate it produces can alter bone marrow hematopoiesis and dendritic cell function, thereby alleviating allergic airway inflammation [16]; gut microbiota-derived desaminotyrosine can enhance type I interferon responses and reduce influenza virus-induced lung injury [17]; acetate can protect airway tight junctions through the G-protein-coupled receptor 43 (GPR43)–AMP-activated protein kinase (AMPK) pathway [18]. In addition to soluble metabolites, the gut microbiota can also influence the migration of group 2 innate lymphoid cells (ILC2) from the intestine to the lung [19]. A recent study further showed that butyrate acts through GPR43 to suppress p38 MAPK/NF-κB signaling and IL-13 production in Tfh13 cells, thereby weakening Tfh13-mediated B-cell responses, reducing IgE^+^ germinal center B cells and plasma cells, and ultimately limiting allergen-specific and anaphylactic IgE production [20]. Accordingly, the role of the gut–lung axis involves multiple levels, including metabolite circulation, the intestinal mucosal barrier, and immune cell reprogramming, providing a potential basis for explaining the distal pulmonary effects of traditional Chinese medicine polysaccharides.

Preclinical studies on the regulation of the gut–lung axis by traditional Chinese medicine polysaccharides have gradually increased in recent years. This narrative mechanistic review compares 16 illustrative preclinical studies (Table 1) that reported an orally administered polysaccharide, a respiratory-disease model, gut microbiota measurements, and a pulmonary outcome. Studies with microbial depletion, transfer, metabolite, or cell-tracing experiments were prioritized. The set is comparative rather than exhaustive and does not support pooled efficacy estimates. Building on the ‘polysaccharides, glycosidic bonds, bacteria, enzymes’ and ‘structure, decoding, transformation, effect’ frameworks [3,8], evidence is organized from polysaccharide structure and microbiota selection to metabolic transformation, cross-organ transmission, and pulmonary effects.

## 2. Traditional Chinese Medicine Polysaccharides Specifically Drive Candidate Gut Bacteria and Their Metabolic Networks

### 2.1. Polysaccharide Structure Is the First Threshold for Screening Candidate Bacteria

The portion of orally administered traditional Chinese medicine polysaccharides that is not fully hydrolyzed by host digestive enzymes can enter the colon and become a carbon source competitively utilized by gut microorganisms. For Bacteroidetes bacteria, the recognition, surface cleavage, oligosaccharide transport, and intracellular degradation of complex polysaccharides are usually coordinated by polysaccharide utilization loci (PULs). In addition to SusC/SusD-like binding-transport proteins, typical PUL can also encode carbohydrate-active enzymes (CAZymes), including glycoside hydrolases (GHs), polysaccharide lyases (PLs), carbohydrate esterases (CEs), carbohydrate-binding modules (CBMs), and sulfatases [1]. Therefore, a match between polysaccharide structure and strain PUL/CAZyme composition may enable utilization and subsequent expansion, although this match has not been tested for most preparation-strain pairs in Table 1, while molecular weight, solubility, and spatial conformation further influence whether the substrate can approach the enzymes on the bacterial cell surface and its degradation rate.

#### 2.1.1. Acidic Polysaccharides

Acidic polysaccharides rich in uronic acid constitute the majority in this statistical collection, including *Atractylodes* polysaccharide ALP, *Houttuynia cordata* polysaccharides HCP and HCPM, *Glycyrrhiza* polysaccharide GCP-2, *Ficus pumila* polysaccharide FPP-1-1, *Cordyceps militaris* polysaccharide CMP, *Ephedra* polysaccharide ESP, and *Tetrastigma hemsleyanum* polysaccharide THP. Among them, the pectin-like characteristics of GCP-2, HCPM, and ESP are the most clearly defined. GCP-2 forms a homogalacturonan (HG)-type main chain with →4)-α-D-GalpA-(1→ and its methyl-esterified residues, and is a typical homogeneous pectin-like acidic polysaccharide; HCPM contains both GalA and Rha as well as arabinan and galactan side chains, exhibiting a more complex branched pectin-like structure; ESP has GalA as the main monosaccharide, and also contains GlcA, Rha, and Ara, presenting overall obvious characteristics of pectin-type polysaccharides. ALP, HCP, FPP-1-1, CMP, and THP all possess relatively clearly defined acidic heteropolysaccharide properties. ALP and HCP contain relatively high proportions of GalA, GlcA, or total uronic acid, and also include various monosaccharides such as Ara, Gal, Rha, Glc, and Man; the uronic acid content of FPP-1-1 reaches 25.14%, but it is dominated by neutral sugars such as Gal and Ara, belonging to a high-molecular-weight, uronic acid-rich complex acidic heteropolysaccharide; CMP is a glucose-dominant acidic glycoprotein polysaccharide, and its 12.94% uronic acid content gives it obvious acidic properties; THP is a highly branched acidic heteropolysaccharide composed of GlcA, Man, Gal, and Ara, with glucuronic acid residues present in both its main chain and side chains.

From the perspective of microbial utilization, these polysaccharides commonly possess uronic acid residues, strong hydrophilicity, and resistance to complete degradation by host digestive enzymes, enabling them to enter the colon relatively intact [37]. HCPM, GCP-2, and ESP have pectin-like features that could be utilized by strains with matching PULs. Known pectin-degradation systems include CE and PL enzymes, GH28, GH105, and GH106, with GH43, GH51, GH53, GH2, and GH35 potentially acting on side chains. These are candidate mechanisms; the necessary PULs, expression patterns, and enzymes have not been verified for most polysaccharide-strain pairs discussed here. The oligosaccharides, acetate, and lactate produced by degradation can also support Lachnospiraceae, *Bifidobacterium*, *Lactobacillus*, and butyrate-producing bacteria, forming a cross-feeding network in which primary degradation and secondary fermentation are connected. In terms of the microbiota responses brought about by oral administration, after intervention with HCPM, GCP-2, ESP, and THP, relatively typical Bacteroidota-related responses appeared, including *Phocaeicola vulgatus*, *Bacteroides caccae*, and other *Bacteroides*. These taxa include strains with diverse PUL repertoires; their enrichment alone does not establish direct utilization of the administered polysaccharide. However, this enrichment did not appear consistently in all studies. After intervention with HCP, FPP-1-1, CMP, and ACP, Bacteroidetes or Muribaculaceae decreased instead. A possible explanation is that PULs have obvious species- and strain-level specificity, and the fact that a certain *Bacteroides* can utilize a polysaccharide does not mean that the entire Bacteroidota will increase. More refined sequencing methods, such as full-length 16S rRNA sequencing or metagenomic sequencing, are needed to further observe changes at the species level [38].

#### 2.1.2. Mannan, Glucan, and Neutral Sugar-Dominant Heteropolysaccharides

Complex heteropolysaccharides dominated by neutral sugars include ACP, AAP, LRP, AABP-60/AABP-80, and APS. ACP is mainly composed of Man, Glc, and Xyl, which together account for nearly 98%, with uronic acid accounting for only 3.246%; its molecular weight reaches 478.604 kDa, and it contains both α and β glycosidic bonds and forms a semi-crystalline dense lamellar structure, belonging to a high-molecular-weight heteropolysaccharide dominated by mannose-glucose-xylose; AAP is mainly composed of Man, and also contains considerable Glc, GlcA, and Xyl, with abundant acetyl groups, making it an acetylated complex heteropolysaccharide dominated by mannan; AABP-60 and AABP-80 are likewise dominated by Man and Glc, with only small amounts of ManA or GulA, and have molecular weights of 56.879 and 357.473 kDa, respectively, belonging to glucomannan-type crude polysaccharide fractions of different molecular weights; LRP is composed of Glc, Ara, and Gal, with Glc as the main component, and contains β-type glycosidic bonds and arabinofuranose structures, overall closer to a glucose-arabinose-galactose-type neutral heteropolysaccharide; APS is composed of Glc, Ara, GlcA, Rha, Man, and Gal, with Glc and Ara being relatively abundant and GlcA content relatively low, and its degradation characteristics are more likely determined by the linkage patterns of mannose, glucose, or related neutral sugar chains.

The microbial utilization mechanisms of such polysaccharides are highly heterogeneous [39]. The Man, Glc, and Xyl structures in ACP, AAP, and AABP may require the joint participation of mannanases, glucanases, xylanases, and corresponding exoglycosidases; LRP and APS may require multiple CAZyme systems due to residues such as Ara, Gal, Rha, and GlcA. In terms of microbial enrichment, these polysaccharides can enrich carbohydrate-utilizing or SCFA-related bacterial groups such as Lachnospiraceae, *Bifidobacterium*, *Akkermansia*, *Lactococcus*, *Oscillospira* and *Coprococcus*, and can also reduce inflammation-related bacterial groups such as *Escherichia-Shigella*, Fusobacteriaceae, and Deferribacteraceae. Their ecological effects are more likely to depend on coordinated degradation by multiple bacterial species rather than being dominated by a single PUL.

#### 2.1.3. Fructans

This category includes FFP/FFP1 and POP. Both are low-molecular-weight β-D-fructans with fructose as the absolute dominant component. The purified fraction FFP1 has a molecular weight of 5.891 kDa, and is mainly composed of linear→1)-β-D-Fruf-(2→linkages, with a degree of polymerization of about 26 and a small amount of terminal Glcp, belonging to a typical linear inulin-type fructan; POP has a molecular weight of about 5.1 kDa, with a β-(2→1)-linked Fruf main chain and β-(2→6)-linked Fruf side chains, and a degree of polymerization of about 31, belonging to a low-molecular-weight fructan with a certain degree of branching.

Compared with high-molecular-weight acidic heteropolysaccharides and complex neutral polysaccharides, fructans have relatively regular structures and lower molecular weights, and are usually more easily utilized by bacterial groups possessing fructan utilization loci, β-fructosidases, or fructan hydrolases. Their primary degradation can release fructo-oligosaccharides and fructose, which then support *Lactobacillus*, *Bacteroides*, Muribaculaceae, and other secondary fermenters [40]. After FFP intervention, increases in Muribaculaceae and some Firmicutes members appeared, accompanied by changes in phenylalanine metabolism; POP increased *Lactobacillus murinus*, Muribaculaceae, and, in fecal microbiota transplantation (FMT) recipients, *Alistipes*, Prevotellaceae_UCG-001, and *L. johnsonii*. The two have similar structures but different microbiota and pulmonary effects, indicating that although the linear or branched degree of fructans affects fermentability, the disease model, initial microbiota, and host immune environment also determine the final response. FFP1 directly inhibited transforming growth factor beta 1 (TGF-β1)/Smad and fibrosis, whereas POP mainly regulated asthma through microbiota-dependent ILC2 migration, also suggesting that fructans can simultaneously possess intestinal ecological effects and different host effector pathways.

#### 2.1.4. Sulfated Polysaccharides

This category includes only EPPs. EPPs are mainly composed of Rha, Xyl, and Glc, with a relatively broad molecular weight distribution, and their most distinctive structural feature is sulfate modification. Unlike the carboxyl negative charge conferred by uronic acid, the anionic properties of EPPs mainly derive from sulfate ester groups, and therefore they cannot simply be classified as pectin-type acidic polysaccharides.

Sulfation modification increases the negative charge, water solubility, and structural complexity of polysaccharides, while also restricting the access of ordinary glycoside hydrolases to the sugar chains. Their complete degradation usually requires sulfatases to first remove sulfate groups, followed by continued cleavage by GH or PL enzymes targeting rhamnose, xylose, and glucose linkages [41]; therefore, the range of bacterial groups capable of directly utilizing EPPs is relatively narrow. After EPP intervention, *Alistipes*, *Lactobacillus*, Lachnospiraceae_UCG-001, and Eubacterium_coprostanoligenes_group increased, while Desulfovibrio and Erysipelotrichaceae decreased, suggesting that they may improve the microbiota through the coordination of specialized degraders and secondary fermenters. However, because existing structural analyses do not indicate sulfate content, substitution positions, main chain linkage patterns, or major molecular weight fractions, EPPs can currently only be defined as sulfated heteropolysaccharides, and it is still insufficient to further establish the correspondence among sulfation sites, sulfatases/PUL, and responsive bacterial groups.

#### 2.1.5. Molecular Weight and Higher-Order Conformation

The polysaccharides involved in existing studies differ significantly in molecular weight, with an overall distribution of approximately 3.52–1290 kDa. Low-molecular-weight fractions mainly include POP (5.1 kDa), FFP1 (5.891 kDa), ESP (about 8.06 kDa), CMP (15.94 kDa), GCP-2 (18.26 kDa), and HCPM (19.1 kDa); medium-molecular-weight polysaccharides include LRP (59.432 kDa), AABP-60 (56.879 kDa), and the major fraction of AAP (137.6 kDa); high-molecular-weight polysaccharides are represented by THP (322.5 kDa), AABP-80 (357.473 kDa), ACP (478.604 kDa), and FPP-1-1 (1290 kDa). In addition, ALP, HCP, and AAP all contain multiple molecular weight fractions, and the molecular weight distribution of EPPs is also relatively broad, indicating that some studies still use polysaccharide systems with a certain degree of polydispersity rather than a single homogeneous fraction. Molecular weight may affect the dissolution, diffusion, retention, and microbial accessibility of polysaccharides in the gastrointestinal tract. Low-molecular-weight polysaccharides generally have better water solubility and lower steric hindrance, and may more easily approach CAZymes on the bacterial surface and be cleaved into transportable oligosaccharides. POP and FFP1 are both fructans of about 5–6 kDa with relatively regular structures, and respectively exhibit regulatory effects on gut-derived ILC2 migration and the phenylalanine metabolic network; ESP is accompanied by increases in multiple SCFAs, suggesting that low-molecular-weight polysaccharides may elicit relatively rapid microbial fermentation responses. High-molecular-weight and highly branched polysaccharides are usually difficult for bacteria to directly take up, and require primary degraders carrying corresponding PULs or extracellular CAZymes to first complete debranching and main chain cleavage on the cell surface, followed by cross-feeding through oligosaccharide release and metabolite exchange. Although FPP-1-1, ACP, and THP reach 1290, 478.604, and 322.5 kDa, respectively, they can still significantly reshape the gut microbiota and alleviate lung injury, indicating that high molecular weight does not constitute a limitation to intestinal ecological effects, but may instead provide a relatively stable ecological niche for specific glycan-utilizing bacteria by increasing intestinal retention time and substrate selectivity.

In addition to molecular weight, the higher-order conformation of polysaccharides further determines the degree of sugar chain exposure and the accessibility of microbial enzymes [42]. ALP exhibits an amorphous, loose, and porous structure, which may facilitate water infiltration and contact between enzymes and sugar chains; FPP-1-1 does not possess a triple-helix conformation, suggesting that its chain segments lack highly ordered aggregation, and even with a relatively high molecular weight, it may still retain certain conformational flexibility. ACP exhibits a semi-crystalline, dense lamellar structure in the solid state, but forms random coils with flexible branches in aqueous solution, reflecting a difference between its solid-state packing and solution conformation, and this conformation may result in relatively slow but sustained microbial degradation. FFP1 exhibits a spherical molecular conformation, while THP is closer to a random coil; their different chain segment folding and hydrodynamic volumes may affect their modes of contact with bacterial surface binding proteins, CAZymes, and host receptors.

In summary, the polysaccharides in the table show clear differences in uronic acid or sulfate content, monosaccharide composition, glycosidic bond types, degree of branching, molecular weight, and spatial conformation. These structural features may constrain microbial access and utilization, but the specific preparation–strain–PUL relationships remain largely untested in the studies reviewed. Matching PUL/CAZyme repertoires are therefore a plausible, rather than generally validated, explanation for selective utilization and cross-feeding. Furthermore, the regulation of gut microbiota by traditional Chinese medicine polysaccharides appears to be understandable as structure-driven redistribution of ecological niches, rather than a fixed promoting effect on a specific beneficial bacterium.

### 2.2. Candidate Primary Degraders and Cross-Feeding in Microbial Networks

When polysaccharides enter the colon, they and their degradation products contribute to microbial nutritional networks. Across the 16 studies, taxonomic responses differed by substrate, model, and baseline microbiota, and no genus changed consistently across all settings. Several studies reported SCFAs or other metabolic outputs, but taxonomic shifts alone do not establish metabolite production or pulmonary mediation. The following sections therefore distinguish putative primary degradation, cross-feeding, and ecological effects from experimentally verified causal links.

#### 2.2.1. Strain-Level Substrate Utilization and Its Evidentiary Limits

As noted in Section 2.1, PUL repertoires and substrate use can vary substantially among strains of the same species. Human-derived Segatella copri isolates, for example, differ in their utilization of xylan, pectin, and β-glucan; disruption of PUL4 impairs barley β-glucan use in some strains, whereas strains lacking PUL4 can use alternative systems [43,44]. Accordingly, differing responses to HCPM, GCP-2, ESP, FFP1, and POP may reflect polysaccharide structure together with basal diet, disease model, and baseline community composition; enrichment patterns alone cannot resolve these contributions.

Some Bacteroides and Phocaeicola strains carry PULs for complex glycans [45], but enrichment alone does not establish utilization of a specific preparation. HCPM and Phocaeicola vulgatus illustrate a candidate route: HCPM was associated with *P. vulgatus* expansion and acetate production, followed by GPR43/JAK2/STAT3-related Th17/Treg regulation, but direct HCPM utilization and microbiota dependence remain untested. These links require in vitro growth and cleavage assays, identification of the relevant PUL and CAZymes, and loss-of-function validation. Thus, candidate primary degraders in this compilation should be treated as hypotheses whose activity depends on polysaccharide structure and the baseline community [46].

#### 2.2.2. Cross-Feeding Converts Primary Degradation Products into Functional Outputs Such as Acetate and Butyrate

Although the microbiota compositions reported in the different studies we compiled differ clearly, multiple studies ultimately converge on the production of acetate, propionate, and butyrate. This is mainly attributed to the fact that primary degraders usually do not exclusively retain all hydrolysis products; degradation enzymes located on the cell surface can release some oligosaccharides and monosaccharides to the extracellular environment, allowing strains lacking complete polysaccharide utilization systems to obtain fermentable substrates. Taking fungal β-glucan as an example, *Bacteroides cellulosilyticus* WH2 can use GH30- and GH157-related enzymes to cleave the intact polysaccharide, and the released gluco-oligosaccharides subsequently support the growth of specific *Bifidobacterium* and Lactiplantibacillus strains, which cannot utilize the original β-glucan when cultured alone [47]. Therefore, the increases in *Bifidobacterium* and *Lactobacillus* observed after CMP, ACP, or AAP intervention may depend on primary degraders such as *Bacteroides* providing substrates.

The next stage of cross-feeding is usually manifested as an organic acid relay. After utilizing oligosaccharides, *Bifidobacterium* and some *Lactobacillus* mainly produce lactate and acetate, and these products can be further utilized by specific butyrate-producing bacteria. Co-culture experiments of isolates have shown that *B. longum* or *B. pseudocatenulatum* produce lactate after utilizing xylan hydrolysates; *Megasphaera indica* can hardly utilize the corresponding sugar substrates when cultured alone, but in the co-culture system it can consume lactate, upregulate genes related to lactate transport and butyrate synthesis, and ultimately increase butyrate production [48]. In this compilation, AAP, ESP, and APS reflect different forms of organic acid relay. After AAP intervention, *Bifidobacterium*, *B. animalis*, and *A. muciniphila* increased, accompanied by elevations in fecal acetate, propionate, butyrate, and other short-chain fatty acids. A more reasonable explanation is that the lactate, acetate, or oligosaccharides released by these bacteria are further utilized by other terminal fermenters, thereby increasing butyrate output. ESP significantly increased multiple cecal short-chain fatty acids and was accompanied by increases in *Butyricicoccus*, Paraprevotella, and *Bacteroides*; APS increased serum acetate, propionate, and butyrate, and showed that propionate and butyrate could inhibit NF-κB activation and M1 polarization in primary alveolar macrophages. The Lachnospiraceae, Lachnospiraceae_NK4A136_group, or related members that appeared in the FPP-1-1, CMP, and ACP studies are more appropriately explained as potential butyrate-producing endpoints rather than the sole primary degraders of the original polysaccharide.

In addition to short-chain fatty acids, the compilation also revealed at least three other types of microbial functional outputs. ALP could restore serum tryptophan and indole-related metabolites, enhance aryl hydrocarbon receptor (AhR) expression in colonic and lung tissues, and antibiotic treatment markedly weakened its protective effects, suggesting that the microbiota-tryptophan metabolism-AhR pathway has relatively strong supporting evidence. After FFP intervention, phenylalanine increased and was correlated with Ruminiclostridium_E and UBA7173; exogenous phenylalanine could also alleviate TGF-β1-induced fibrotic phenotypes, supporting phenylalanine as a potential effector metabolite. The THP study involved multiple metabolic pathways including tryptophan, branched-chain amino acids, porphyrins, fatty acid amides, and glycerophospholipids.

In summary, the functional outputs formed after transformation of traditional Chinese medicine polysaccharides by the microbiota are mainly short-chain fatty acids, but are not limited to short-chain fatty acids. Different bacteria can maintain similar fermentation functions through shared substrates and continued utilization of intermediate metabolites, while microbiota composition more strongly influences the composition of specific products [49]. Existing studies provide the most consistent support for short-chain fatty acids, while changes in tryptophan, phenylalanine, and lipid metabolism indicate that polysaccharides may also induce broader metabolic regulation, but the relevant evidence mostly remains at the correlational level. Overall, the effects of polysaccharides reflect changes in the overall metabolic capacity of the microbiota, and their specific outputs are jointly influenced by polysaccharide structure, the baseline microbiota, and disease state.

#### 2.2.3. Taxonomic Shifts, Barrier Responses, and Possible Colonization Resistance

Another important common feature of microbiota changes in the studies compiled here is the accompanying decline in opportunistic pathogens or inflammation-related taxa, including decreases in Desulfovibrionaceae/*Desulfovibrio* after ALP and EPPs intervention, decreases in *Escherichia-Shigella* after GCP-2 and LRP intervention, decreases in Proteobacteria, Fusobacteria, and Fusobacteriaceae after AAP intervention, and decreases in Campylobacteria and Gram-negative bacterial phenotypes after AABP intervention. These taxonomic shifts are compatible with, but do not demonstrate, stronger colonization resistance. Short-chain fatty acid-mediated local acidification can inhibit some Enterobacteriaceae bacteria, while butyrate can also promote colonic epithelial β-oxidation and peroxisome proliferator-activated receptor γ (PPAR-γ) signaling, reducing the availability of respiratory electron acceptors such as luminal oxygen and nitrate, thereby limiting the expansion of bacterial groups dependent on aerobic or nitrate respiration [50,51,52]. In addition, diverse commensal bacteria can form nutritional blockade by jointly covering the carbon and nitrogen sources required by pathogens, and microbial community combinations with complementary resource utilization profiles can significantly enhance colonization resistance [53,54]. This provides a reasonable explanation for the decline in Enterobacteriaceae-related taxa after GCP-2, AAP, and LRP intervention. More direct evidence comes from the mucosal barrier. GCP-2 restored intestinal tight junction proteins while reducing serum lipopolysaccharide (LPS), and inhibited pulmonary TLR4/NF-κB signaling; EPPs upregulated colonic ZO-1, Claudin-1, and innate immunity activator (INAVA); AABP improved goblet cells and colonic mucosal damage, and increased MUC2, ZO-1, Occludin, and Claudin-1 expression; ALP simultaneously improved intestinal and pulmonary barriers and restored tryptophan-AhR signaling. Together, these observations support a plausible metabolite–barrier–lung pathway, but do not establish its continuity in each intervention.

**Figure 1:**
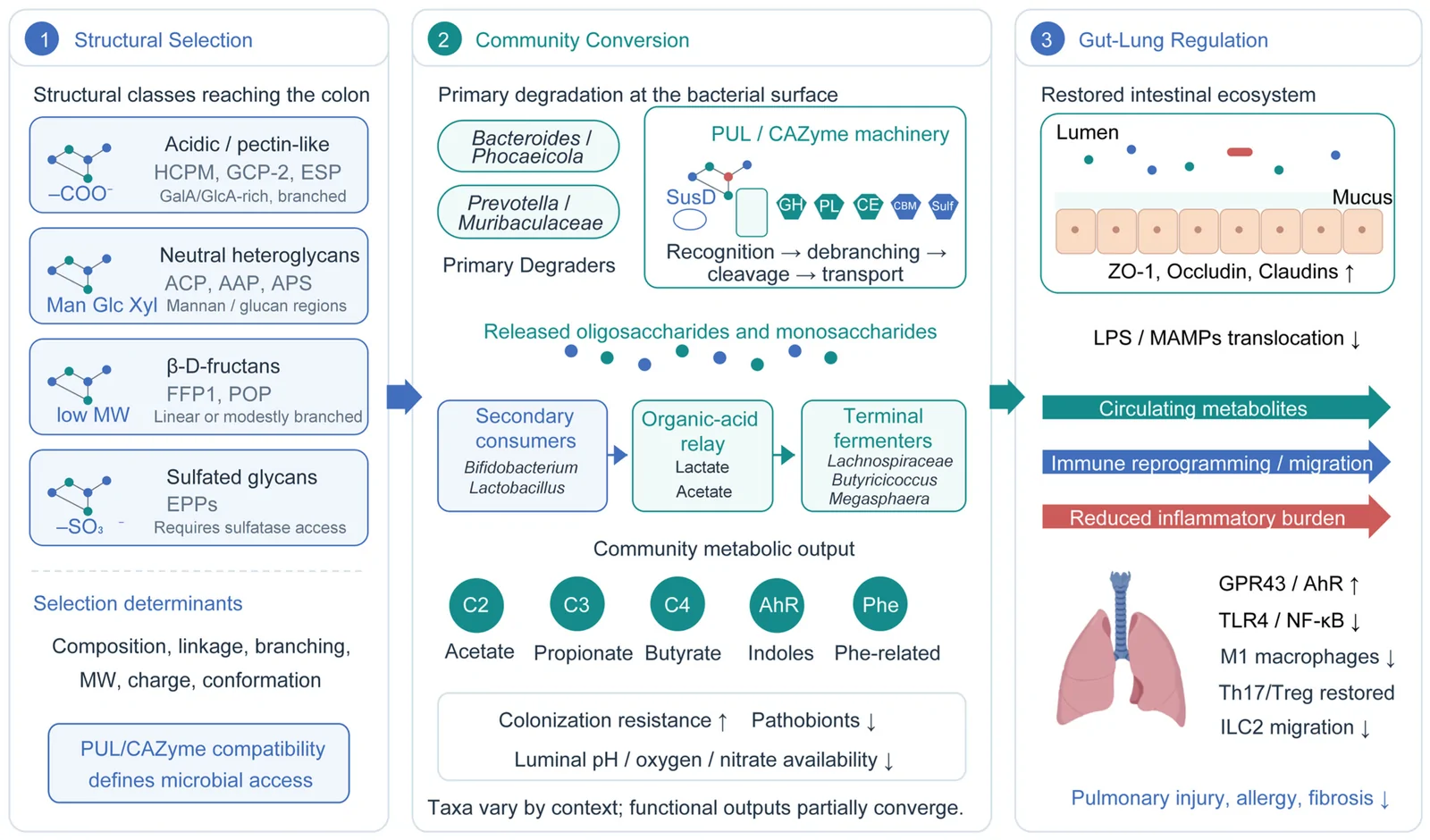
Cascade process of structural specificity of natural polysaccharides driving gut microbiota remodeling and metabolite generation. PUL, polysaccharide utilization locus; CAZyme, carbohydrate-active enzyme; GH, glycoside hydrolase; PL, polysaccharide lyase; CE, carbohydrate esterase; CBM, carbohydrate-binding module; Sulf, sulfatase; Phe, phenylalanine; GPR43, G-protein-coupled receptor 43; AhR, aryl hydrocarbon receptor; TLR4, Toll-like receptor 4; NF-κB, nuclear factor kappa B; Th17, T helper 17 cell; Treg, regulatory T cell; ILC2, group 2 innate lymphoid cell. Created with BioRender.com.

In summary, the decline in opportunistic pathogens after traditional Chinese medicine polysaccharide intervention is more likely to originate from changes in the intestinal ecological environment rather than direct inhibition of these bacteria by the polysaccharides. The utilization of nutritional substrates by commensal bacteria can alter luminal pH, redox state, and the availability of electron acceptors, while mucosal barrier repair and alleviation of local inflammation also weaken the expansion advantage of some inflammation-related bacteria. Under the combined action of these factors, the growth of groups such as *Desulfovibrio* and *Escherichia-Shigella* is restricted. Therefore, the improvement of the gut microbiota by polysaccharides is manifested not only as increases in some commensal bacteria, but may also accompany changes in colonization resistance and mucosal barrier markers, which may thereby reduce the entry of intestinal inflammatory signals such as LPS into the circulation, as shown in Figure 1.

## 3. Polysaccharide-Driven Microbiota and Metabolic Networks Influence Pulmonary Pathology via the Gut–Lung Axis

### 3.1. Microbiota-Derived Metabolites Mediate Gut–Lung Signaling

Microbiota-derived metabolites provide the most direct biochemical interface between polysaccharide fermentation and host responses. In the studies included in this article, SCFAs are the most frequently detected metabolites, but their mechanistic interpretation requires distinguishing luminal production, portal and systemic exposure, receptor binding, intracellular HDAC inhibition, and responses of lung target cells [13,55,56]. Related studies have shown that acetic acid, propionic acid, and butyric acid differ in abundance, first-pass utilization, receptor preference, and HDAC inhibition potency [56]; therefore, total fecal SCFAs should not be regarded as a unified surrogate for gut–lung signaling.

#### 3.1.1. SCFAs Activate Receptor-Dependent Signaling Pathways

Acetic acid and propionic acid can effectively activate FFAR2/GPR43, while propionic acid and butyric acid are the main ligands of FFAR3/GPR41; butyric acid can also activate HCAR2/GPR109A [57,58]. Receptor activation can alter Gi/o- or Gq-related cAMP, calcium, and MAPK signaling; further, its biological output depends on receptor abundance, cell type, ligand concentration, and disease context. Related studies have shown that in colitis, arthritis, and asthma models, Gpr43-deficient mice exhibit excessive or unresolving inflammatory responses [59]. Some studies have further found that SCFAs maintain the abundance and suppressive function of colonic Tregs through FFAR2, while this effect of propionic acid disappears in *Ffar2*-deficient Treg cells [60]. Among the polysaccharide studies involved in this article, acetic acid derived from HCPM-*Phocaeicola vulgatus* is associated with intestinal GPR43/JAK2/STAT3 signaling and Th17/Treg regulation [23], while butyric acid in asthma models inhibits Tfh13-related p38 MAPK/NF-κB signaling through GPR43 [20]. In contrast, the involvement of GPR41/43 and GPR109A after LRP treatment still requires further experimental support [33].

#### 3.1.2. SCFAs Regulate Immune Function Through Epigenetic Mechanisms

SCFAs can also act through chromatin-level mechanisms after cellular uptake, especially HDAC inhibition. Butyric acid usually has the strongest HDAC inhibitory activity, propionic acid is weaker, and acetic acid has limited activity at comparable concentrations [61,62,63]. In mice, butyric acid increases histone H3 acetylation at the *Foxp3* promoter and conserved noncoding regions, promotes extrathymic or colonic Treg differentiation, and alters inflammatory gene expression in dendritic cells [61,62]. Studies have shown that propionic acid reduces HDAC6/9 expression and increases H3K9 acetylation in colonic Treg cells in an FFAR2-dependent manner [60], indicating that receptor-dependent and epigenetic mechanisms may converge. In human naive CD4+ T cells, butyric acid and propionic acid enhanced the differentiation and suppressive capacity of induced Tregs in vitro, accompanied by changes in histone H4 acetylation at Treg-related loci [64]. These human cell-derived findings support biological plausibility, but do not establish in vivo pulmonary or clinical effects.

Epigenetic responses are also cell- and context-dependent. In intestinal macrophages, butyric acid suppresses LPS-induced inflammatory mediators through HDAC inhibition [65]. In another context, butyric acid confers an antimicrobial program on macrophages and improves resistance to intestinal pathogens [66]. SCFAs can also support both effector and regulatory T cell programs through HDAC inhibition and the mTOR-S6K pathway [67]. These apparently different effects indicate that dose, cell activation state, tissue microenvironment, and microbial context largely determine the functional outcome.

#### 3.1.3. Circulating Microbial Metabolites Influence Pulmonary Target Cells

After epithelial absorption, acetic acid, propionic acid, and butyric acid produced by colonic fermentation first enter the portal vein, but the proportions of the three reaching the peripheral circulation are not the same. Human isotope tracer studies have shown that the systemic utilization rates of colonically administered acetic acid, propionic acid, and butyric acid are approximately 36%, 9%, and 2%, respectively, indicating that most butyric acid and propionic acid are utilized in first-pass effects through the intestine and liver, while acetic acid more readily forms detectable peripheral exposure [68]. APS provides relatively complete evidence of systemic dissemination. Oral APS enriched potential short-chain fatty acid-related bacteria such as *Oscillospira*, *Akkermansia*, and *Coprococcus*, while increasing serum acetic acid, propionic acid, and butyric acid levels; propionic acid and butyric acid in turn reduced the expression of Cd86, IL-1β, IL-6, and TNF-α in primary alveolar macrophages and inhibited NF-κB p65 phosphorylation. The level of evidence from other studies included in this article is relatively limited. AAP and ESP respectively increased multiple short-chain fatty acids in feces or cecal contents, but did not simultaneously measure concentrations in the portal vein, peripheral blood, or lung tissue. In addition, a study on *Ficus pumila* polysaccharide (FP-P) showed that in smoke-exposed COPD mice, total cecal short-chain fatty acids increased by 41.90%, among which propionic acid and butyric acid increased by 45.45% and 23.41%, respectively, while acetic acid showed no significant change; PICRUSt2 also predicted enhancement of butyrate synthesis and carbohydrate degradation pathways [69]. SCFAs may also affect lung pathology through direct lung cell responses and indirect immune programming. In differentiated human bronchial epithelial cells, propionic acid and butyric acid increased transepithelial electrical resistance and restored barrier damage induced by IL-4, IL-13, or house dust mite extract, while restoring ZO-1 expression and ERK1/2 and JNK signaling [70]. Together with propionic acid-FFAR3-mediated bone marrow myeloid remodeling [16], the effects of SCFAs on alveolar macrophages [35], and butyric acid-GPR43 regulation of the Tfh13-IgE axis [20], these findings identify several pathways through which polysaccharide fermentation may affect the lungs.

In addition to short-chain fatty acids, the studies included in this article also suggest that other circulating metabolites may participate in cross-organ transmission. FFP intervention was accompanied by elevated serum phenylalanine, and exogenous phenylalanine can reduce TGF-β1-induced fibronectin and vimentin expression in vitro, providing a potential link between phenylalanine metabolism and pulmonary fibrosis; ALP restored serum tryptophan and indole-related metabolic profiles and enhanced AhR expression in colon and lung tissues; THP corrected multiple metabolic pathways in plasma, including tryptophan, branched-chain amino acids, and glycerophospholipids. Bile acid receptors may constitute another branch. Studies have shown that Bletilla striata polysaccharide BSP can enrich *Bacteroides* intestinalis; colonization by this bacterium enhanced the protective effect of BSP against smoke-induced COPD and upregulated NR1H4 and its encoded FXR, while reducing NR1H4 expression weakened the effects of BSP and B. intestinalis [71]. This study relatively strongly supports the functional connection of BSP-B. intestinalis-NR1H4/FXR.

Preclinical respiratory models further demonstrate disease-specific effects. A high-fiber diet protected mice from influenza infection by shaping the hematopoiesis of Ly6C^+^ patrolling monocytes and supporting CD8+ T cell metabolism [72], while SCFAs ameliorated antibiotic-associated allergic lung inflammation by altering dendritic cell and T cell responses [73]. Conversely, influenza-associated gut microbiota dysbiosis reduced SCFA production and impaired the antimicrobial activity of alveolar macrophages, thereby increasing susceptibility to secondary Streptococcus pneumoniae infection [74]. Therefore, in gut–lung axis research, the measurement of SCFAs in the portal vein, peripheral blood, and lung tissue should be combined with receptor blockade or cell-specific deletion, HDAC activity, site-specific histone acetylation assays, and disease-related functional endpoints.

### 3.2. Intestinal Barrier Repair Limits Gut-Derived Inflammatory Signaling

Gut microbiota metabolites are a key link connecting the gut–lung axis. However, related signals such as short-chain fatty acids, tryptophan derivatives, and bile acids can form controlled gut–lung information transfer only under the premise that the intestinal mucosa maintains selective permeability. Once the mucus layer, epithelial junctions, or mucosal immune barrier are damaged, what enters the circulation is no longer limited to small-molecule metabolites at physiological concentrations, but may also include LPS, lipoteichoic acid, peptidoglycan fragments, bacterial DNA, bacterial extracellular vesicles, and even intact bacterial cells. At this point, the intestine shifts from homeostatic metabolism to continuous input of inflammatory stimuli to the lungs. Therefore, intestinal mucosal barrier repair is an important effector checkpoint that determines whether microbiota remodeling can be translated into lung-protective effects. The intestinal mucosal barrier consists of multiple interconnected layers. MUC2 secreted by goblet cells forms the colonic mucus layer, isolating most bacteria from the epithelial surface; after *Muc2* deletion, bacteria can directly contact the epithelium and enter the crypts [75]. SIgA enhances mucosal immune exclusion by limiting bacterial adhesion, coating bacteria, and cross-linking dividing daughter bacteria [76]. Beneath it, proteins such as Claudin, Occludin, and ZO-1 jointly maintain the selectivity of the epithelial paracellular pathway [77]. The intestinal vascular barrier beneath the epithelium also constitutes a subsequent checkpoint limiting the systemic dissemination of bacteria and their products [78]. Polysaccharide-mediated barrier repair can be understood as the combined restoration of mucus, SIgA, epithelial junctions, and vascular restriction. For example, ALP, GCP-2, EPPs, and AABP all provide varying degrees of barrier evidence. ALP simultaneously improved intestinal and pulmonary barrier-related proteins, and its barrier and lung-protective effects were significantly weakened after antibiotic treatment, indicating that this effect depends on the microbiota. EPPs upregulated colonic ZO-1, Claudin-1, and INAVA, while alleviating H5N1-induced intestinal and lung tissue damage; AABP-60 and AABP-80 restored the expression of colonic MUC2, ZO-1, Occludin, and Claudin-1, and improved crypt and goblet cell damage, providing relatively direct histological evidence for the synchronous restoration of the mucus layer and tight junctions. GCP-2 intervention was accompanied by restoration of barrier proteins, reduced serum LPS, and inhibition of pulmonary TLR4/NF-κB signaling. In addition, studies on Poria cocos polysaccharide (PCP) further showed that oral PCP can restore intestinal MUC2, SIgA, Occludin, and Claudin-1 in mice infected with multidrug-resistant Klebsiella pneumoniae, reducing lung bacterial burden and TNF-α and IL-1β in BALF; in antibiotic-induced pseudo-germ-free mice, the above effects were significantly weakened [79]. Mechanistically, microbiota-derived butyric acid may be an important mediator linking polysaccharide metabolism and barrier repair. Classic studies have shown that butyric acid can promote the relocation of ZO-1 and Occludin to cell junctions by activating AMPK, increasing transepithelial electrical resistance and reducing paracellular flux, without necessarily requiring a significant increase in the total expression of these proteins [80]. Among the studies included in this article, AAP, APS, and ESP directly detected increases in short-chain fatty acids such as butyric acid, while CMP, ACP, and FPP-1-1 mainly inferred related effects based on the enrichment of potential butyrate-producing taxa such as Lachnospiraceae. Furthermore, SCFA-dependent barrier effects are not limited to tight junction relocation. In colonic epithelial cells, microbiota-derived butyric acid increased oxygen consumption and stabilized hypoxia-inducible factor, thereby enhancing barrier-protective transcription; depletion of butyrate-producing microbiota impaired this response [81]. This finding enhances the biological plausibility of the metabolite-barrier pathway. Barrier repair directly affects the pathological process of the gut–lung axis, as described below.

#### 3.2.1. Barrier Repair Limits Systemic Dissemination of Gut-Derived PAMPs

After intestinal barrier disruption, pathogen-associated molecular patterns (PAMPs), such as LPS, lipoteichoic acid, and peptidoglycan, as well as intact bacterial cells, can enter distant organs through the portal-systemic circulation or mesenteric lymphatics [82]. In patients with sepsis and acute respiratory distress syndrome, more gut-derived taxa can be detected in the lung microbiota; viable gut-associated bacteria also appear in the lungs of experimental septic mice, and these changes correlate with the degree of alveolar inflammation [83]. A tracer study provided more direct evidence. After antifungal drug-induced intestinal fungal dysbiosis, Escherichia coli abnormally expanded with increased intestinal permeability; the researchers subsequently detected pre-colonized GFP-labeled Escherichia coli in blood and lung tissue [84]. This result indicates that intestinal ecological imbalance can not only increase circulating LPS levels, but may also allow intact bacterial cells to cross the barrier. Therefore, it can be inferred that the reduction of *Escherichia-Shigella* after GCP-2 and LRP treatment, and the reduction of Desulfovibrionaceae or Desulfovibrio after ALP and EPPs treatment, may help reduce the gut-derived PAMP burden, but this inference still needs to be confirmed by strain labeling, portal vein sampling, and cross-organ tracing.

#### 3.2.2. Reducing Gut-Derived Inflammatory Exposure Prevents Abnormal Pulmonary Immune Activation

High-load PAMP exposure caused by persistent barrier disruption can place alveolar and interstitial macrophages in an abnormally pre-activated state [85]. LPS enhances inflammatory gene transcription through the TLR4-MyD88-NF-κB pathway and can provide a priming signal for the NLRP3 inflammasome; when the lungs are subsequently stimulated by bacteria, viruses, or particulate matter, pre-activated macrophages are more likely to release large amounts of IL-1β, TNF-α, and IL-6, thereby promoting neutrophil recruitment, alveolar-capillary barrier disruption, and tissue edema. GCP-2 reduced serum LPS and inhibited pulmonary TLR4/NF-κB, while AAP downregulated pulmonary TLR4 and MyD88; both can be explained by barrier repair further reducing gut-derived PAMPs and thereby reducing macrophage inflammatory responses. It should be noted that the goal of barrier repair is not to completely cut off microbial signaling between the intestine and lungs [86]. Antibiotic clearance of intestinal commensal bacteria weakens the phagocytic and bactericidal capacity of alveolar macrophages, increasing lung and blood bacterial burden in mice with Klebsiella pneumoniae pneumonia; appropriate supplementation with TLR4 ligands can partially restore pulmonary defense [87]. Therefore, gut-derived signals have obvious dose and context dependence. Specifically, physiological levels of stimulation help maintain basal pulmonary immune surveillance, while persistent high-load exposure after barrier disruption leads to excessive inflammation. The potential mode of action of traditional Chinese medicine polysaccharides is to restore gut-derived PAMP exposure to a homeostatic range, rather than completely silencing pattern recognition pathways such as TLR4.

#### 3.2.3. Barrier Repair Interrupts the Bidirectional Amplification of Intestinal and Pulmonary Inflammation

The impact of intestinal inflammation on the lungs is a bidirectional process, and respiratory infection can also reverse-damage intestinal ecology and the mucosal barrier [88]. After intranasal influenza virus infection in mice, obvious intestinal immune damage can be induced even when the virus is not detected in the small intestine [89]. The mechanism involves lung-derived CCR9^+^CD4^+^ T cells migrating to the small intestine and releasing IFN-γ, subsequently altering the gut microbiota, promoting epithelial IL-15 expression and local Th17 polarization, and ultimately mediating intestinal damage through IL-17A [89]. This can form a continuous positive feedback loop, in which pulmonary infection or inflammation causes circulating cytokines and immune cell migration, thereby inducing gut microbiota dysbiosis and barrier disruption; increased gut-derived PAMPs, bacterial components, and inflammatory mediators re-enter the circulation, further activating pulmonary macrophages and vascular endothelium, and aggravating lung tissue damage. ALP, EPPs, and AABP can simultaneously improve intestinal and pulmonary pathology, suggesting that they may weaken this feedback at multiple nodes. However, synchronous recovery of both organs cannot determine the temporal order of effects: it may be that the polysaccharide first repairs the intestine and reduces intestinal inflammatory input, or it may be that intestinal damage secondarily improves after pulmonary inflammation is alleviated. Future studies can combine continuous time-point sampling, intestinal epithelium-specific injury or repair models, barrier re-disruption, portal vein and mesenteric lymphatic tracing, and FMT experiments to clarify the initiation site and transmission direction of polysaccharide effects [90].

### 3.3. Immune Cell Reprogramming Links Intestinal Signals with Pulmonary Responses

Intestinal mucosal repair can reduce the continuous input of microbial components and inflammatory mediators from the intestine into the circulation, but it is still insufficient to explain why traditional Chinese medicine polysaccharides can separately improve viral pneumonia, allergic airway inflammation, sterile acute lung injury, and infection-related severe pneumonia. A deeper effect may lie in the ability of polysaccharides and their microbial metabolites to alter the differentiation direction, tissue origin, migration and homing, and effector programs of specific immune cells, thereby ultimately manifesting changes in intestinal ecology as adjustments in pulmonary immune status. Here, immune cell reprogramming refers to changes in lineage differentiation, migration, homing, local reactivation, and effector state [91]; chromatin-level SCFA effects have been separately discussed in Section 3.1.2. In the summary of this article, HCP and HCPM mainly involve Th17/Treg balance; POP points to the migration of gut-derived ILC2s to the lungs; CMP, ACP, and ESP provide a metabolic basis to be verified for butyric acid regulation of the Tfh13-IgE axis; and APS and THP involve alveolar macrophages, complement-neutrophil, and immunothrombotic responses. These effects can be explained in specific cell lineages, sites of origin, and disease contexts, as described below.

#### 3.3.1. The Th17/Treg Axis Links Intestinal Immune Regulation with Pulmonary Inflammation

Th17 cells and Tregs share partially overlapping differentiation contexts, but tend toward inflammatory amplification and immune tolerance, respectively. During influenza virus infection, persistently enhanced Th17 responses can promote neutrophil recruitment and alveolar barrier damage through IL-17A; Treg cells help limit immune-mediated tissue destruction [92]. Therefore, evaluating the regulation of the Th17/Treg axis by polysaccharides requires not only observing the cell ratio in the lungs, but also determining whether changes first occur in the intestine or the lungs, and distinguishing cell differentiation, migration, and local reactivation. Mechanistic studies on HCP provide tissue-level temporal evidence for this question. Oral HCP first restored the Th17/Treg balance in gut-associated lymphoid tissues, and only afterward did corresponding changes appear in the lungs, whereas intravenous administration could not produce equivalent protective effects. HCP simultaneously reduced CCL20 expression in lung tissue and adjusted the proportions of CCR6^+^Th17 and CCR6^+^Treg cells; in vitro experiments showed that it inhibited STAT3 phosphorylation and Th17 differentiation, and enhanced STAT5 phosphorylation and Treg differentiation. In RORγt- or *Foxp3*-deficient mice, the lung-protective effects of HCP were significantly weakened, indicating that the relevant cell lineages are an important basis for its immune effects [7]. Subsequent studies further found that after antibiotic clearance of the microbiota, the regulatory effect of HCP on the CCR6^+^Th17/CCR6^+^Treg ratio disappeared, while fecal microbiota transplantation from HCP donors could reconstruct part of the immune protective effect in recipients, supplementing evidence of microbiota dependence. HCPM studies further linked Th17/Treg changes with specific strains and metabolites. Compared with the control polysaccharide AAP01-1, which lacks lung-protective effects, HCPM selectively promoted *Phocaeicola vulgatus* and intestinal luminal acetic acid production; acetic acid enema activated GPR43 in intestinal tissue, inhibited JAK2/STAT3 signaling, and restored Th17/Treg balance, ultimately accompanied by alleviation of H1N1 lung injury. The resulting “HCPM-P. vulgatus-intestinal acetic acid-GPR43/JAK2/STAT3-Th17/Treg” chain is an example with relatively high mechanistic support among the studies included in this article. Notably, this study did not detect a stable increase in serum acetic acid, indicating that microbial metabolites may first reshape T cell status locally in the intestinal mucosa, and then affect the lungs through lymphocyte output, chemokine axis changes, or other circulating signals, without necessarily manifesting as a sustained increase in peripheral blood concentration. In addition, another study on H1N1 and methicillin-resistant Staphylococcus aureus coinfection found that a homologous *Houttuynia cordata* polysaccharide could inhibit the overactivated intestinal C3a/C5a-NLRP3 inflammatory module and correct Treg/Th17 imbalance; improvement in intestinal damage preceded pulmonary recovery [93]. This study suggests that complement and inflammasomes may constitute another immunoregulatory pathway beyond the acetic acid-GPR43 pathway. Future studies can combine intestinal photoconversion labeling, gut–lung paired TCR clonal analysis, stable isotope tracing, and T cell-specific gene knockout [94,95,96] to distinguish the mode of metabolite entry into the lungs from the mode of intestinal T cell reprogramming followed by migration.

#### 3.3.2. Gut-Derived ILC2 Migration Contributes to Allergic Airway Inflammation

In allergic airway inflammation, group 2 innate lymphoid cells (ILC2s) can rapidly produce IL-5 and IL-13 in response to IL-25, IL-33, and TSLP, promoting eosinophil infiltration, goblet cell metaplasia, and mucus hypersecretion [97]. In previous studies, lung ILC2s were mostly regarded as locally expanded tissue-resident cells [98], but POP studies suggest that under disease conditions, lung ILC2s may also be affected by input from gut-derived cells. In an OVA-induced allergic airway inflammation model, antibiotic treatment significantly weakened the ameliorative effects of POP on lung histopathology, serum IgE, type 2 cytokines, and the numbers of Th2 and ILC2 cells, while fecal microbiota transplantation from POP donors could reconstruct part of the protective effect. Further adoptive transfer and in vivo tracing of gut-derived ILC2s showed that OVA stimulation promoted the accumulation of labeled intestinal ILC2s in the lungs, while POP or POP donor FMT reduced this migration, along with reducing IL-33 in lung tissue and the numbers of ILC2 and Th2 cells. Therefore, POP may limit the spread of intestinal type 2 immune responses to the lungs by altering cell tissue distribution and lung homing. At the same time, after POP treatment, Clostridia_UCG-014 and Rikenellaceae_RC9 decreased, while Muribaculaceae, Prevotellaceae_UCG-001, and some *Lactobacillus* increased [31]. Future studies can use endogenous photoconversion, parabiosis, and tissue-specific fate tracing, combined with chemokine receptor blockade and strain colonization experiments, to analyze the actual contribution of gut-derived ILC2s and the molecular basis of their migration.

#### 3.3.3. The Tfh13-IgE Axis Links Microbial Butyrate to Allergen-Specific Humoral Responses

Elevated IgE in allergic asthma is not merely the result of a general Th2 response. Tfh13 is a type of follicular helper T cell that simultaneously expresses BCL6, GATA3, IL-4, and IL-13, and can promote germinal center B cells to produce high-affinity IgE with strong sensitizing capacity, representing an important link between T cell differentiation and allergic humoral immunity [99]. Studies have shown that among the major short-chain fatty acids, butyric acid has the most prominent inhibitory effect on Tfh13 and allergic airway inflammation. Single-cell transcriptome and immunophenotypic analyses showed that butyric acid mainly reduced Tfh13 in mediastinal lymph nodes and weakened their interactions with germinal center B cells and plasma cells. Mechanistically, butyric acid depends on GPR43 to inhibit p38 MAPK and NF-κB p65 activation, thereby reducing the expression of IL-13, GATA3, and BCL6; GPR43 antagonism weakens this effect. Dietary interventions that increase colonic butyrate production also reduced Tfh13 and sensitizing IgE in OVA, house dust mite, and humanized asthma models [20]. This progress provides new potential mechanistic directions for CMP, ACP, and ESP in the studies included in this article. CMP, ACP, and ESP may affect the Tfh13-IgE axis by remodeling the butyrate production network, among which the metabolic evidence for ESP is relatively more direct, but a causal loop has not yet been established. Future studies can, in addition to detecting serum total IgE and Th2 cytokines, simultaneously measure butyric acid in feces, portal vein, and peripheral blood, analyze Tfh13, IgE^+^ germinal center B cells, and IgE^+^ plasma cells in mediastinal lymph nodes, and evaluate antigen specificity and actual sensitizing capacity.

#### 3.3.4. Microbial Metabolites Regulate Macrophage and Neutrophil Effector Functions

Alveolar macrophages and neutrophils not only undertake pathogen recognition and clearance, but can also cause bystander damage through cytokines, reactive oxygen species, proteases, extracellular traps, and coagulation responses [100]. APS provides relatively direct evidence that microbial metabolites regulate alveolar macrophages. APS enriched potential short-chain fatty acid-related bacteria such as *Oscillospira*, *Akkermansia*, and *Coprococcus*, and increased serum acetic acid, propionic acid, and butyric acid levels; propionic acid and butyric acid reduced the expression of Cd86, IL-1β, IL-6, and TNF-α in primary alveolar macrophages and inhibited NF-κB p65 phosphorylation, while in animal lung tissue, proinflammatory markers decreased and repair-related markers increased. This suggests that SCFAs may lower the threshold for excessive alveolar macrophage responses to LPS stimulation. Regarding neutrophils, THP reduced MPO and Ly6G signals in a two-hit severe pneumonia model, and reduced the deposition of C3 and the platelet marker CD42b, while improving pulmonary hemorrhage, apoptosis, and microthrombus-related changes. These results suggest that THP may affect immunothrombotic processes involving complement, neutrophils, and platelets. It should be noted that under infectious conditions, the role of neutrophils is dual-sided. In the context of antibiotic control of Streptococcus pneumoniae, reducing neutrophils improved oxygenation, pulmonary edema, and epithelial damage, but was accompanied by a certain increase in alveolar bacterial burden [100]; another study showed that TLR5 agonist combined with antibiotics could reshape the composition of early neutrophil subsets, enhancing functional groups with phagocytic and antimicrobial properties, thereby improving host-directed therapeutic effects. The aforementioned indole-3-acetic acid study also supports this point: IAA enhances neutrophil ROS production through AhR, which can reduce bacterial dissemination but simultaneously aggravate lung tissue damage [42]. Therefore, when judging the role of traditional Chinese medicine polysaccharides in infectious lung injury, evaluation of neutrophil chemotaxis, phagocytosis, and pathogen killing functions should be retained as much as possible.

### 3.4. Disease Context Determines the Dominant Gut–Lung Mechanism

The above three mechanisms can operate in parallel or sequentially. Their relative contributions are determined by gut-derived signals and the pre-existing pulmonary microenvironment; therefore, it cannot be assumed that the same changes in SCFA production produce the same outcomes in allergy, viral infection, bacterial pneumonia, sterile injury, COPD, and fibrosis [101].

**Figure 2:**
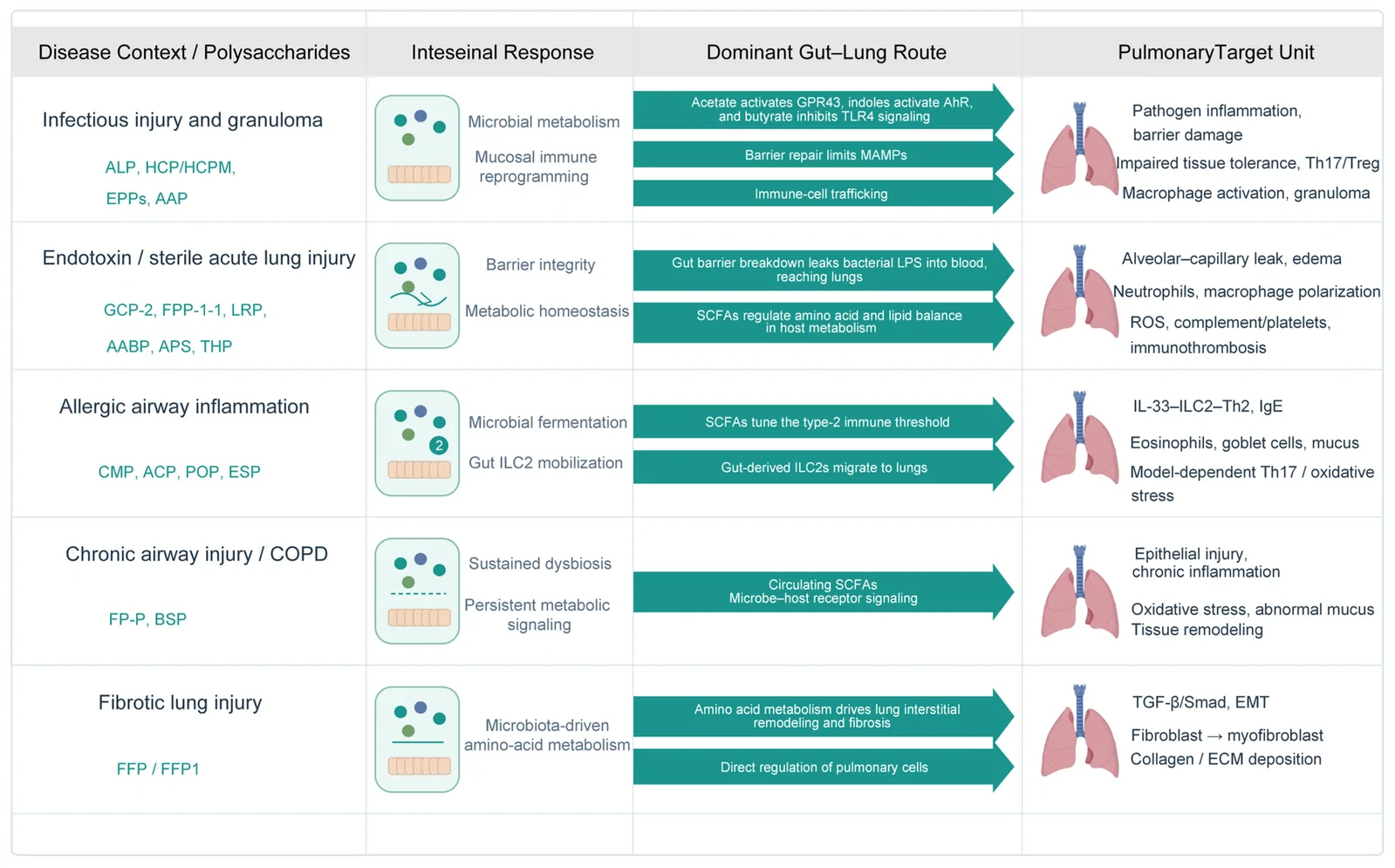
Differing underlying mechanisms in various lung diseases. IgE, immunoglobulin E; IL-33, interleukin-33; ILC2, group 2 innate lymphoid cell; LPS, lipopolysaccharide; LRP, Lycium ruthenicum polysaccharide; MAMPs, microbe-associated molecular patterns; POP, Polygonatum odoratum fructan; ROS, reactive oxygen species; SCFAs, short-chain fatty acids; TGF-β, transforming growth factor beta; THP, Tetrastigma hemsleyanum polysaccharide; Th2, T helper 2 cells; Th17, T helper 17 cells; TLR4, Toll-like receptor 4; Treg, regulatory T cells. Created with BioRender.com.

Although traditional Chinese medicine polysaccharides can usually induce gut microbiota remodeling, barrier repair, and microbial metabolic changes, these gut-derived changes do not produce the same downstream effects in all lung diseases. The pre-existing pathological environment of the lung constitutes a second layer of screening: infectious diseases emphasize the balance between pathogen control and tissue damage, acute sterile injury mainly manifests as amplification of innate immune cascades, allergic diseases center on type 2 immune deviation, and fibrotic diseases ultimately point to interstitial cell activation and extracellular matrix deposition. Therefore, the gut–lung axis effects of traditional Chinese medicine polysaccharides should be understood in combination with disease-specific effector cells, signaling pathways, and pathological endpoints. See Figure 2. Experimental SCFA effects illustrate this context dependence. FFAR2 signaling supports inflammation resolution in multiple inflammatory models [59], SCFAs can reduce allergic dendritic cell/Th2 responses [73], and dietary fiber can support antiviral CD8+ T cell metabolism [72]. At the same time, the absence of SCFAs during influenza impairs antibacterial macrophage function and promotes secondary Streptococcus pneumoniae infection [74]. Therefore, lower cytokine concentrations alone are not a sufficient endpoint: studies should evaluate inflammatory indicators together with pathogen burden, barrier integrity, tissue damage, and related pulmonary effector cell functions.

Overall, the difference among diseases lies not mainly in whether traditional Chinese medicine polysaccharides can regulate the microbiota, but in what kind of pulmonary effect the microbiota changes are ultimately translated into. Infectious diseases emphasize rebalancing pathogen clearance, mucosal immunity, and tissue tolerance; endotoxic or sterile acute injury mainly depends on reducing the gut-derived inflammatory burden and blocking innate immune cascades; allergic airway inflammation highlights type 2 immune reprogramming and gut-derived immune cell migration; chronic airway injury may depend more on sustained metabolite-receptor signaling; fibrotic lung injury further points to interstitial remodeling and extracellular matrix deposition. Future studies can focus on the key effector cells and irreplaceable pathological endpoints of various diseases, and establish experiments involving microbial clearance, specific bacterial colonization, metabolite supplementation, receptor blockade, and cell migration tracing, in order to truly determine the relative contribution of each gut–lung axis pathway in different diseases.

## 4. Discussion and Conclusions

Starting from the in vivo process of orally administered traditional Chinese medicine polysaccharides, this narrative review examines selected preclinical evidence on their regulation of the gut–lung axis. Together, these studies motivate a proposed sequence from polysaccharide structure and potential microbial utilization to cross-feeding, metabolites, host responses, and pulmonary outcomes; however, the full sequence has not been verified for each preparation. This framework helps explain why some macromolecular polysaccharides can still produce distal effects despite limited exposure of their parent forms in lung tissue, and can also accommodate the differences among different polysaccharides, baseline microbiota, and pulmonary pathological environments. From this perspective, the gut–lung axis effects of traditional Chinese medicine polysaccharides originate from the joint action of polysaccharide structure, microbial functional reserves, and host disease state, and changes in a single bacterial genus or a certain pulmonary inflammatory pathway can only reflect one link in this process. For attribution, microbiota-dependent effects require a perturbation such as depletion or transfer that changes the pulmonary outcome; microbiota-associated effects show parallel gut and lung changes without that test. Direct host–cell activity should be considered separately. For example, purified FFP1 inhibited TGF-β1-induced cellular changes in vitro [28].

A relatively stable understanding obtained in this review is that differences in taxonomic composition of the microbiota and a certain degree of convergence in metabolic functions can exist simultaneously. In the table, the directions of change of groups such as Muribaculaceae, *Bacteroides*, and *Lactobacillus* are not entirely consistent across different studies, yet multiple interventions jointly point to short-chain fatty acid production, barrier repair, and mucosal immune homeostasis. This phenomenon is consistent with the functional redundancy and cross-feeding of the gut microbiota. Different primary degraders can release similar oligosaccharides or organic acids, and intermediate products can be further utilized by different terminal fermenters. Pooled studies on multiple types of dietary fiber also show that substrate type and pre-intervention microbiota composition can jointly determine butyrate production potential and the main contributing bacterial groups [102]. At the same time, enhanced polysaccharide fermentation and elevated short-chain fatty acids need to be judged in combination with specific disease contexts. High-level studies have shown that inulin can activate FXR, IL-33, and ILC2-related responses through microbiota bile acid metabolism, increase intestinal and pulmonary eosinophils, and aggravate allergic inflammation [103]. Recent studies have further found that a pectin-rich high-fiber diet and the propionate it produces can remodel alveolar macrophage metabolism and alleviate sterile lung injury, but weaken pulmonary defense and aggravate infection in bacterial infection models [104]. These results suggest that the direction of action of the same metabolite can change with concentration, phase of action, target cells, and type of pulmonary pathology. Infectious diseases especially require balancing pathogen clearance and tissue tolerance; excessive inhibition of macrophage or neutrophil responses may reduce tissue injury, but may also affect pathogen control. Allergic diseases require attention to the specific effects of metabolites on ILC2, Tfh13, IgE, and epithelial alarmins. Therefore, the evaluation of the functional outputs of traditional Chinese medicine polysaccharides should cover metabolite profiles, host targets, and disease-related endpoints, and should not be judged solely by changes in total short-chain fatty acids or common inflammatory factors. From the perspective of evidence hierarchy, studies on traditional Chinese medicine polysaccharides regulating the gut–lung axis have begun to move from parallel associations toward continuous causal verification. HCP used antibiotic depletion and FMT; HCPM linked a candidate bacterium and acetate supplementation to GPR43-related responses without the same gut-dependence test; and POP combined antibiotic depletion, FMT, and evidence of gut-derived ILC2 migration. These studies test different links, but do not all establish a continuous causal chain. Antibiotics and FMT can support microbiota dependence, but the former can simultaneously alter host immunity and metabolism, and the latter can also transfer metabolites, phages, and other biological components. A more ideal evidence pathway can include standardization of polysaccharide structure, direct utilization by strains and verification of key PUL, stable isotope metabolic tracing, zonal detection in the portal vein and lung tissue, immune cell fate tracking, and cell-specific receptor knockout, and ultimately be implemented in disease-related functional endpoints.

Recent studies have also expanded the understanding of cross-organ transmission. The gut microbiota can shape the basal immune state of the lung before infection. For example, segmented filamentous bacteria in the intestine can functionally program alveolar macrophages, enhancing their complement production, phagocytosis, and antiviral capacity [105]. Immune cell migration atlases established based on photoconversion animals and single-cell sequencing indicate that multiple types of colonic immune cells can leave the intestine dependent on S1P signaling and exhibit selective distribution in different peripheral inflammatory tissues [106]. On the other hand, Eggerthella lenta and its related metabolite TUDCA, which are enriched in bronchiectasis patients, can inhibit AMPK activation and energy production in neutrophils, reduce the clearance of Pseudomonas aeruginosa, and thereby aggravate pulmonary infection [107]. These findings indicate that gut–lung information transmission includes not only soluble metabolites entering the circulation, but also immune cell migration and functional preprogramming of bone marrow and lung-resident cells. Its biological consequences have clear context dependence. Future infection models should simultaneously evaluate pathogen load, phagocytic killing, animal survival, and lung tissue injury; allergy models should strengthen indicators such as antigen-specific IgE, Tfh13, and ILC2; fibrosis studies should focus on lung function, fibroblast status, and extracellular matrix remodeling. Only in this way can general anti-inflammatory changes be distinguished from effects that truly improve the disease process. Clinical extrapolation of existing evidence still needs to remain cautious. The studies summarized in this article are almost all derived from animal models, and there are considerable differences in polysaccharide preparation, dosage, administration period, basal diet, animal strain, and microbiota detection methods. The diet and environment of experimental animals are highly controlled, whereas human populations are subject to multiple influences including age, geography, daily diet, medication use, underlying diseases, and previous antibiotic exposure. Recent human dietary fiber intervention studies have shown that pre-intervention dominance of Prevotella or *Bacteroides* can significantly influence microbiota responses [108]; a randomized study involving 802 prediabetic subjects also found that the overall population did not achieve consistent improvement in the primary endpoint, while some baseline metabolic and microbiota subtypes exhibited relatively clear responses [109]. Although these results come from metabolic diseases, they have direct implications for the translation of traditional Chinese medicine polysaccharides. Candidate polysaccharides are more suitable for development as microecological substrates with differences in applicable populations, and potential responder populations can be screened by combining baseline metagenomics, PUL and CAZyme characteristics, in vitro fecal fermentation, and metabolite production capacity. Clinical studies should also clarify dosage, fermentation tolerance, interactions with basal diet and commonly used drugs, and monitor the expansion of opportunistic pathogens, production of harmful metabolites, and gastrointestinal adverse reactions.

Interpretation of the animal evidence also depends on model and reporting quality. OVA allergy, LPS injury, influenza, silicosis, and bacterial infection differ in initiating insults and clinical relevance. Cross-study comparisons should check sample size, animal sex, randomization, blinding, cage and diet, antibiotic exposure, baseline microbiota, sequencing resolution, polysaccharide purity and batch characterization, dose, and independent replication. These items were not assessed systematically here, so the grades above describe mechanistic tests and are not risk-of-bias scores. Interpretation of SCFA-centered mechanisms should also consider co-exposure to other dietary and herbal constituents. Polyphenols can reshape the microbiota and SCFA output, while microbial transformation generates phenolic acids that may converge with SCFAs on HDAC and inflammatory signaling. Evidence from allergy research suggests that oligosaccharide and polyphenol exposures can jointly influence microbial and immune development, although findings for human milk oligosaccharides cannot be directly transferred to traditional Chinese medicine polysaccharides [110]. In vitro comparisons further show that butyrate is a more potent HDAC inhibitor than propionate and most tested polyphenol-derived metabolites, while p-coumaric acid, 3-(4-hydroxyphenyl)propionate, and caffeic acid also inhibit nuclear HDAC activity [63]. These observations support potential interaction at the level of microbiota metabolism and host signaling, but do not demonstrate in vivo synergy. As some of the preparations summarised in Table 1 are not chemically pure, residual polyphenolic compounds or other small molecules may have a potential impact on the microbial community and lung function. Future studies should report polysaccharide purity and total phenolic/flavonoid content, control the basal diet, and use purified fractions or factorial co-administration designs before attributing an SCFA-mediated effect to the polysaccharide alone.

Overall, existing preclinical evidence supports the intestine as an important site of action for orally administered traditional Chinese medicine polysaccharides to produce distal pulmonary effects. The selective utilization determined by polysaccharide structure, microbial cross-feeding, metabolic functional output, barrier homeostasis, and immune information transmission jointly constitute the biological basis of their regulation of the gut–lung axis. Potentially selective utilization, cross-feeding, metabolites, barrier responses, and immune signaling together provide a testable framework for gut–lung effects; their relative contributions remain preparation- and model-dependent. Among them, a few studies have formed relatively complete causal chains, while most candidate polysaccharides are still at the stage of accumulating mechanistic clues. In future evaluations of the research value of traditional Chinese medicine polysaccharides, emphasis can be placed on whether their structures are stable and controllable, whether there is direct evidence of microbial transformation, whether functional outputs can be repeated across cohorts and models, and whether these changes can improve key outcomes matched to specific pulmonary diseases. On this basis, traditional Chinese medicine polysaccharides regulating the gut–lung axis have the potential to gradually develop from descriptive microbiota associations into microecological intervention strategies with analyzable structures, verifiable mechanisms, and identifiable applicable populations.

## Figures and Tables

**Table 1:** Preclinical Evidence on TCM Polysaccharides, Gut Microbiota Changes, and Pulmonary Outcomes.

Disease and Model	TCM Polysaccharide	Structural Features	Gut Microbiota Changes	Major Pulmonary Effects	Gut–Lung Evidence and Proposed Mechanism	Ref.
*Staphylococcus aureus*-induced acute lung injury in mice	*Atractylodes lancea* polysaccharides (ALP)	Water-soluble acidic polysaccharides; total carbohydrate 79.53%, uronic acid 10.58%; two peaks at approximately 83.66 and 3.52 kDa, with 3.52 kDa dominant; Man, GlcN, Rha, GlcA, GalA, Glc, Gal, and Ara at 5.01:2.15:5.99:5.77:20.16:18.10:15.90:26.90; predominantly β-glycosidic linkages; amorphous, loose, porous structure.	Increased *Limosilactobacillus* and decreased Desulfovibrionaceae.	Reduced lung-index elevation, inflammatory-cell abnormalities, and histopathology; lower pulmonary IL-1β and TNF-α; higher lung and intestinal barrier-protein expression.	ALP altered microbiota composition and serum tryptophan- and indole-related metabolite profiles and increased colonic and pulmonary AhR expression. Antibiotic depletion markedly weakened intestinal and pulmonary protection.	[21]
H1N1-induced pneumonia in mice	*Houttuynia cordata* polysaccharides (HCP)	Water-soluble acidic polysaccharides; total carbohydrate 78.74%, uronic acid 31.9%; four peaks at 228,559, 108,043, 57,623, and 33,113 Da; Man, Rha, GlcA, GalA, Glc, Gal, Xyl, and Ara at 6.54:9.30:2.84:15.32:18.66:27.88:5.64:13.82.	Changed the microbiota composition of antibiotic-treated recipients, with decreased Proteobacteria and Bacteroidetes/*Bacteroides*; increased unclassified Lachnospiraceae; accompanied by higher acetate.	Reduced pulmonary edema and parenchymal injury; decreased total and migratory Th17 cells and increased total and migratory Treg cells in the lung.	HCP lowered the CCR6+ Th17/CCR6+ Treg ratio in conventionally colonized mice, but not after antibiotic depletion. Fecal microbiota transplantation (FMT) from HCP-treated donors reproduced the immune changes in recipients.	[22]
H1N1-induced acute lung injury in mice	*Houttuynia cordata* pectin-like polysaccharide (HCPM)	Homogeneous acidic pectin-like polysaccharide, 19.1 kDa; backbone of 1,3,6-β-Manp, 1,4-α-GalpA, 1,2-α-Rhap, and 1,2,4-α-Rhap; glucan, arabinan, and galactan side chains attached mainly at C-3 of 1,3,6-β-Manp or C-4 of 1,2,4-α-Rhap.	Increased *Phocaeicola vulgatus*, *Akkermansia muciniphila*, and *Bacteroides caccae*; corrected H1N1-associated increases in Lachnospiraceae, Eubacterium, and *Oscillibacter*.	Improved lung index and histopathology; lowered TNF-α, IL-6, IFN-γ, and IL-17A; restored IL-10; reduced migratory CCR6+ Th17 cells and increased migratory CCR6+ Treg cells.	HCPM interacted with *P. vulgatus* to generate luminal acetate. Acetate activated GPR43, suppressed JAK2/STAT3 signaling, reduced the intestinal Th17/Treg ratio, and reduced H1N1 lung injury from the intestinal side.	[23]
LPS-induced acute lung injury in mice	*Glycyrrhiza* polysaccharide (GCP-2)	Homogeneous acidic pectin-like polysaccharide, 18.26 kDa; predominantly GalA; homogalacturonan-type chains containing -4)-α-D-GalpA-(1- and -4)-α-D-GalpA-6-OMe-(1-, with minor Rha, Ara, Gal, GlcA, Glc, and Man.	Decreased Proteobacteria and *Escherichia-Shigella*; increased Bacteroidota/Bacteroidetes, *Lactobacillus*, *Bifidobacterium*, Prevotellaceae, and Muribaculaceae.	Lowered lung wet/dry ratio, serum LPS, histopathology score, and F4/80+ macrophage infiltration; reduced IL-1β, IL-6, TNF-α, TLR4/NF-κB/p-p65 signaling, and oxidative stress; restored occludin, ZO-1, and claudins.	GCP-2 altered microbiota composition and lowered gut-derived endotoxin, followed by suppression of pulmonary TLR4/NF-κB activation, inflammation, oxidative stress, and gut and lung barrier injury.	[24]
LPS-induced acute lung injury in mice	*Ficus pumila* polysaccharide (FPP-1-1)	Homogeneous acidic polysaccharide; total carbohydrate 98.4%, uronic acid 25.14%; 1290 kDa; Rha, Man, GlcA, Glc, GalA, Gal, Ara, and Fuc at 5.55:1.79:1.05:3.67:1.00:49.52:13.43:1.10; pyranose and α-glucopyranosidic features; no triple helix.	Increased Firmicutes, *Lactobacillus*, *Lachnoclostridium*, Prevotellaceae_UCG-001, Clostridia, Lachnospirales, Lachnospiraceae, and the Lachnospiraceae_NK4A136 group; decreased Bacteroidota, Proteobacteria, Muribaculaceae, *Dubosiella*, *Helicobacter*, Oscillospiraceae, and Clostridia_UCG-014.	Lowered wet/dry ratio; reduced alveolar hemorrhage, collapse, neutrophil infiltration, and septal thickening; decreased bronchoalveolar lavage fluid (BALF) and lung TNF-α and IL-6; increased IL-10; inhibited ERK1/2, p38, and NF-κB p65 phosphorylation.	The reported microbiota changes occurred alongside reduced pulmonary MAPK/NF-κB signaling. Prevotellaceae_UCG-001 and *Alloprevotella* correlated positively with IL-10 and negatively with IL-6, TNF-α, p-ERK, and p-p65; Muribaculaceae and *Dubosiella* showed the opposite pattern. Lachnospiraceae and *Alloprevotella* are potential butyrate producers.	[25]
Ovalbumin (OVA)-induced allergic asthma in mice	*Cordyceps militaris* polysaccharide (CMP)	Homogeneous acidic glycoprotein polysaccharide; total carbohydrate 66.49%, uronic acid 12.94%; 15.94 kDa; Glc, Man, Gal, Xyl, Ara, and GlcA at 81.25:21.96:13.88:3.92:3.58:1.00; α-pyranose configuration.	Increased Lachnospiraceae and the Firmicutes/Bacteroidetes ratio; decreased Bacteroidetes and Muribaculaceae.	Lowered lung index and serum IgE; reduced epithelial shedding, alveolar-wall thickening, and inflammatory infiltration; decreased TNF-α, IL-4, IL-5, IL-6, IL-13, and IL-17A; increased IFN-γ; activated Nrf2/HO-1/SOD1/SOD2 and inhibited NF-κB/p65.	CMP reduced duodenal villus injury and altered microbiota composition. *Akkermansia* and *Alistipes* correlated positively with IFN-γ and negatively with IgE and type 2 cytokines. Enriched Lachnospiraceae may contribute butyrate or reactive sulfur species that activate Nrf2 and inhibit NF-κB; this remains inferential.	[26]
OVA-induced allergic asthma in mice	*Auricularia cornea* ‘Yumuer’ polysaccharide (ACP)	Homogeneous acidic heteropolysaccharide; total carbohydrate 91.74%, uronic acid 3.246%; 478.604 kDa; Man 48.34%, Glc 35.26%, and Xyl 14.35%, with minor Gal, Fuc, GlcA, GalA, and Ara; α- and β-linkages; semicrystalline dense lamellae in the solid state and a flexibly branched random coil in water.	Increased Lachnospiraceae, *Lactococcus*, and the Firmicutes/Bacteroidetes ratio; decreased Bacteroidetes, Muribaculaceae, Bacteroidaceae, and Deferribacteraceae.	Lowered lung index, serum IgE, and OVA-specific IgE; reduced alveolar epithelial hyperplasia, wall thickening, atrophy, and inflammatory infiltration; decreased type 2/17 cytokines; increased IFN-γ; activated Nrf2/HO-1/SOD1/SOD2 and inhibited NF-κB/p65, iNOS, and COX-2.	ACP improved small-intestinal villus and mucosal injury. Lachnospiraceae and *Lactococcus* correlated negatively, whereas Deferribacteraceae, Muribaculaceae, and Bacteroidaceae correlated positively, with type 2/17 inflammatory indices. Predicted sulfur metabolism and MAPK changes paralleled pulmonary Nrf2/NF-κB signaling.	[27]
SiO2-induced silicosis in mice	Farfarae Flos polysaccharides (FFP/FFP1)	Crude FFP: total carbohydrate 85.21%, mainly <30 kDa. Purified FFP1: homogeneous inulin-type fructan, total carbohydrate 100%, 5.891 kDa; Fru and Glc at 94.4:5.6; mainly linear -1)-β-D-Fruf-(2-, degree of polymerization approximately 26, with minor terminal Glcp-(1-; globular conformation.	Increased Patescibacteria, Firmicutes_B, *Dubosiella*, UBA7173, Muribaculaceae, and Ruminiclostridium_E; decreased *Ligilactobacillus*.	Improved respiratory rate and lung appearance; reduced alveolar collapse, septal thickening, inflammation, and collagen deposition; lowered inflammatory and Ashcroft scores, serum TGF-β1, and pulmonary collagen I, fibronectin, vimentin, and α-SMA. FFP1 inhibited TGF-β1-induced EMT, fibroblast-to-myofibroblast transition, and Smad2/3 phosphorylation in vitro.	FFP altered microbiota composition and serum metabolite profiles, increasing phenylalanine while decreasing phenylpyruvate and phenylacetylglycine. Phenylalanine correlated with Ruminiclostridium_E and UBA7173, and exogenous phenylalanine reduced TGF-β1-induced fibronectin and vimentin. Purified FFP1 also directly inhibited TGF-β1/Smad-mediated EMT and fibroblast transition.	[28]
*Propionibacterium acnes*-induced sarcoidosis-like pulmonary granulomas in mice	*Auricularia auricula* polysaccharides (AAP)	Three molecular-weight fractions, with a major 137.6 kDa fraction; Fuc, Rha, Ara, Gal, Glc, Xyl, Man, and GlcA at 2.48:0.09:0.61:5.58:20.36:12.17:43.47:15.25; dominated by Man, Glc, GlcA, and Xyl; rich in acetyl groups.	Increased Firmicutes, Actinobacteria, Actinomycetia, Bifidobacteriales, *Bifidobacterium*, *B. animalis*, and *Akkermansia*; decreased Bacteroidetes, Proteobacteria, TM7, Fusobacteria, *Mycoplasma*, *Sutterella*, *Cetobacterium*, and Fusobacteriaceae.	Reduced surface nodules, alveolar-wall fusion, uneven alveolar size, inflammatory infiltration, and dense granulomas; lowered pulmonary TNF-α, IL-1β, IL-6, IL-8, TLR4, and MyD88.	AAP enhanced butyrate metabolism and increased fecal acetate, propionate, butyrate, valerate, and isovalerate, with the largest rise in butyrate. *Bifidobacterium*, *B. animalis*, *A. muciniphila*, and *Lactobacillus helveticus* correlated positively with butyrate, whereas butyrate correlated negatively with inflammatory factors, TLR4, and MyD88. Docking predicted binding between butyrate and TLR4.	[29]
H5N1 influenza infection in mice	*Enteromorpha* polysaccharides (EPPs)	Sulfated polysaccharides composed mainly of Rha, Xyl, and Glc, with a broad molecular-weight distribution.	Increased *Alistipes*, *Enterococcus*, the Eubacterium_coprostanoligenes group, ASF356, Lachnospiraceae_UCG-001, *Lactobacillus*, Lactobacillales, and *Turicibacter*; decreased Desulfobacterota/*Desulfovibrio*, Erysipelotrichaceae, *Roseburia*, and *Mucispirillum*.	Lowered pulmonary H5N1 load; reduced diffuse injury, alveolar-wall destruction, hemorrhage, and inflammatory infiltration; decreased pulmonary CCL2, IFN-γ, IL-17, and TNF-α; restored IL-10.	EPPs reduced inflammation and histopathology in both lung and colon; increased colonic ZO-1, claudin-1, and INAVA; increased *Alistipes* and *Lactobacillus* while suppressing *Desulfovibrio*; accompanied by changes in amino sugar/nucleotide sugar, pyruvate, and amino acid metabolism.	[30]
OVA-induced allergic airway inflammation in mice	*Polygonatum odoratum* polysaccharide (POP)	Low-molecular-weight fructan; total carbohydrate 95.3%; 5.1 kDa; Fru and Glc at 30:1; predominantly (2-1)-β-D-Fruf backbone with (2-6)-linked β-D-Fruf side chains; no acetylation; degree of polymerization approximately 31.	Increased *Lactobacillus murinus* and Muribaculaceae; decreased Clostridia_UCG-014 and Rikenellaceae_RC9. POP-donor FMT recipients also showed increases in Muribaculaceae, *Alistipes*, Prevotellaceae_UCG-001, and *L. johnsonii* and decreases in Clostridia_UCG-014, Rikenellaceae_RC9, and *Bacteroides acidifaciens.*	Reduced lung enlargement, parenchymal and alveolar-wall thickening, goblet-cell metaplasia, mucus, and inflammatory infiltration; lowered serum IgE, BALF eosinophils, IL-4, IL-5, and IL-13; reduced pulmonary IL-33, Th2 cells, and ILC2s.	Antibiotics weakened pulmonary, IgE, eosinophil, cytokine, Th2, and ILC2 responses to POP; POP-donor FMT reconstructed part of the protection. Gut-derived ILC2 transfer and in vivo tracking showed that OVA promoted intestinal ILC2 migration to the lung, whereas POP and POP-donor FMT restrained it.	[31]
PM2.5 plus OVA-induced exacerbated asthma-like disease in mice	*Ephedra sinica* polysaccharide (ESP)	Acidic heteropolysaccharide, approximately 8.06 kDa; mainly GalA (57.9%) and GlcA, with minor Ara, Glc, Rha, Gal, and Man; pectin-like features.	Decreased Firmicutes, Actinobacteria, *Enterococcus*, and *Ruminococcus*; increased Bacteroidetes, Proteobacteria, *Bacteroides*, *Lactobacillus*, *Prevotella*, *Butyricicoccus*, and *Paraprevotella*.	Lowered lung index and BALF total inflammatory cells, eosinophils, neutrophils, monocytes, and lymphocytes; reduced airway infiltration, alveolar-wall thickening, goblet-cell hyperplasia, and mucus; lowered serum IgE, TNF-α, IL-1β, and IL-6 and pulmonary NF-κB, TNF-α, and IL-1β transcripts.	ESP increased cecal acetate, propionate, butyrate, isobutyrate, valerate, isovalerate, and isocaproate, with butyrate most abundant. *Parabacteroides*, *Blautia*, and *Butyricimonas* correlated positively with several SCFAs. Predicted carbohydrate metabolism increased alongside lower pulmonary NF-κB signaling and inflammatory mediators.	[32]
LPS-induced acute lung injury in mice	*Lycium ruthenicum* Murr. polysaccharide (LRP)	Water-extracted, alcohol-precipitated heteropolysaccharide, 59.432 kDa; D-Glc, L-Ara, and D-Gal at 3.426:1.400:1.000; pyranose and β-glycosidic features, with signals consistent with arabinofuranose rings and glucuronic acid carbonyl groups.	Increased Bacillota, Bacteroidota, and *Bacteroides*; decreased Pseudomonadota, *Escherichia-Shigella*, and *Eisenbergiella*.	Lowered wet/dry ratio; reduced alveolar hemorrhage, collapse, neutrophil infiltration, and septal thickening; decreased TNF-α and IL-6 levels in bronchoalveolar lavage fluid (BALF) and lung tissue; increased IL-10; inhibited ERK1/2, p38, and NF-κB p65 phosphorylation.	Changes in microbiota composition and pulmonary outcomes occurred in parallel. A proposed mechanism is microbial production of acetate, propionate, and butyrate from LRP, followed by signaling through GPR41/43, GPR109A, and NLRP3; this causal chain was not directly tested.	[33]
Lipoteichoic acid-induced pulmonary inflammation in mice	*Anemarrhena asphodeloides* polysaccharides (AABP-60 and AABP-80)	Water-extracted, alcohol-precipitated crude fractions. AABP-60: 56.879 kDa; ManA, Man, and Glc at 0.033:1.000:0.665. AABP-80: 357.473 kDa; GulA, Man, Glc, and Gal at 0.499:11.521:5.903:1.000.	Both fractions decreased Bacteroidetes and Campylobacteria and increased Firmicutes. AABP-60 decreased Muribaculaceae and increased Lactobacillaceae, Lachnospiraceae, and Rikenellaceae; AABP-80 decreased Muribaculaceae and Rikenellaceae and increased Lactobacillaceae and Prevotellaceae. Predicted Gram-negative and pathogenic phenotypes declined.	Reduced alveolar disorganization, lymphocyte and macrophage infiltration, congestion, and edema; restored alveolar structure; lowered serum TNF-α and the abnormal LTA-induced increase in IL-10, with AABP-80 generally more active.	AABP altered microbiota composition, improved colonic mucosal, crypt, and goblet-cell injury, and increased colonic MUC2, ZO-1, occludin, and claudin-1.	[34]
LPS-induced inflammatory lung injury in mice	*Astragalus membranaceus* polysaccharides (APS)	Water-extracted and alcohol-precipitated, followed by DEAE-cellulose water elution; Man, Rha, GlcA, Glc, Gal, and Ara at 1.647:1.893:2.317:7.251:1.000:4.351.	Increased Firmicutes, *Oscillospira*, *Akkermansia*, and *Coprococcus*; decreased Bacteroidetes and *Prevotella*.	Reduced alveolar destruction, wall thickening, erythrocyte and inflammatory-cell infiltration, wet/dry ratio, BALF cell number and protein, neutrophils, and lung/serum myeloperoxidase (MPO); lowered *Icam*-1, IL-1β, IL-6, TNF-α, *Ccl2*, and *Cxcl1*; shifted macrophages away from M1 markers toward M2 markers; inhibited NF-κB p65 phosphorylation.	APS increased the relative abundance of several putative SCFA-producing taxa and increased serum acetate, propionate, and butyrate. Propionate and butyrate reduced IL-1β, IL-6, TNF-α, and *Cd86* and inhibited NF-κB p65 phosphorylation in primary alveolar macrophages.	[35]
Two-hit severe pneumonia induced by intratracheal LPS followed by intraperitoneal LPS in mice	*Tetrastigma hemsleyanum* polysaccharide (THP)	Acidic heteropolysaccharide; total carbohydrate 83.5%; 322.5 kDa; Rha, Ara, Gal, Glc, Man, and GlcA at 1.928:7.771:23.379:1.000:13.660:17.614. Backbone with alternating -4)-α-Galp-(1-, -2)-α-Manp-(1-, and -4)-β-GlcpA-(1-; branches at C-3 or C-4 of -2,3)-Manp-(1- and -2,3,4)-Manp-(1-; terminal α-Araf, α-Galp, β-GlcpA(4-OMe), and -4)-β-GlcpA(6-OMe) side-chain motifs; near-random-coil conformation.	Increased Bacteroidota, Muribaculaceae, Odoribacter, Bacteroides, Oscillospiraceae, and Rikenellaceae_RC9; corrected an abnormal increase in Lactobacillus.	Lowered wet/dry ratio and BALF protein; reduced edema, hemorrhage, necrosis, inflammation, and apoptosis; decreased pulmonary F4/80, MPO, C3, CD42b, and Ly6G; suppressed macrophage infiltration, neutrophil recruitment, immunothrombosis, inflammatory mediators, and TLR4/NF-κB/COX-2 signaling.	THP altered microbiota composition and plasma metabolite profiles, including lysophospholipids, fatty acid amides, and central carbon metabolism, including tryptophan, branched-chain amino acid, porphyrin, and glycerophospholipid pathways. These changes accompanied inhibition of pulmonary TLR4/NF-κB/COX-2 signaling.	[36]

Glcp: D-glucopyranose; Manp: D-mannopyranose; Galp: D-galactopyranose; GalpA: D-galactopyranuronic acid; GlcpA: D-glucopyranuronic acid; Fruf: D-fructofuranose; Araf: L-arabinofuranose; Rhap: L-rhamnopyranose and ManA: D-mannuronic acid.

## Data Availability

No new data were created or analyzed in this study. Data sharing is not applicable to this article.

## Abbreviations

The following abbreviations are used in this manuscript:

AABP	Anemarrhena asphodeloides polysaccharides
AAP	Auricularia auricula polysaccharides
ACP	Auricularia cornea ‘Yumuer’ polysaccharide
AhR	Aryl hydrocarbon receptor
ALP	Atractylodes lancea polysaccharides
AMPK	AMP-activated protein kinase
APS	Astragalus membranaceus polysaccharides
Ara	Arabinose
Araf	Arabinofuranose
BALF	Bronchoalveolar lavage fluid
BCL6	B-cell lymphoma 6
BSP	Bletilla striata polysaccharide
C3	Complement component 3
CAZymes	Carbohydrate-active enzymes
CBMs	Carbohydrate-binding modules
CCL	C-C motif chemokine ligand
CCR	C-C motif chemokine receptor
CD	Cluster of differentiation
CEs	Carbohydrate esterases
CMP	*Cordyceps militaris* polysaccharide
COPD	Chronic obstructive pulmonary disease
COX-2	Cyclooxygenase-2
ECM	Extracellular matrix
EMT	Epithelial–mesenchymal transition
EPPs	Enteromorpha polysaccharides
ERK1/2	Extracellular signal-regulated kinase 1/2
ESP	*Ephedra* sinica polysaccharide
F4/80	Murine macrophage marker F4/80
FFAR2/3	Free fatty acid receptors 2 and 3
FFP	Farfarae Flos polysaccharides
FFP1	Purified Farfarae Flos polysaccharide fraction 1
FMT	Fecal microbiota transplantation
FP-P	*Ficus pumila* polysaccharide
FPP-1-1	*Ficus pumila* polysaccharide FPP-1-1
Fruf	Fructofuranose
Fuc	Fucose
FXR	Farnesoid X receptor
Gal	Galactose
GalA	Galacturonic acid
Galp	Galactopyranose
GalpA	Galactopyranuronic acid
GATA3	GATA-binding protein 3
GCP-2	Glycyrrhiza polysaccharide GCP-2
GFP	Green fluorescent protein
GHs	Glycoside hydrolases
Glc	Glucose
GlcA	Glucuronic acid
GlcN	Glucosamine
Glcp	Glucopyranose
GlcpA	Glucopyranuronic acid
GPR	G protein-coupled receptor
GulA	Guluronic acid
H1N1	Influenza A virus subtype H1N1
HCP	*Houttuynia cordata* polysaccharides
HCPM	*Houttuynia cordata* pectin-like polysaccharide
HO-1	Heme oxygenase-1
ICAM-1	Intercellular adhesion molecule 1
IFN-γ	Interferon-γ
IgA	Immunoglobulin A
IgE	Immunoglobulin E
IL	Interleukin
ILC2s	Group 2 innate lymphoid cells
INAVA	Innate immunity activator
JAK2	Janus kinase 2
LPS	Lipopolysaccharide
LRP	Lycium ruthenicum polysaccharide
LTA	Lipoteichoic acid
Ly6G	Lymphocyte antigen 6 complex locus G6D
MAMPs	Microbe-associated molecular patterns
Man	Mannose
ManA	Mannuronic acid
Manp	Mannopyranose
MAPK	Mitogen-activated protein kinase
MPO	Myeloperoxidase
MUC2	Mucin 2
MyD88	Myeloid differentiation primary response 88
NF-κB	Nuclear factor-κB
NLRP3	NOD-like receptor family pyrin domain-containing 3
NR1H4	Nuclear receptor subfamily 1 group H member 4
Nrf2	Nuclear factor erythroid 2-related factor 2
OMe	O-methyl group
OVA	Ovalbumin
PAMPs	Pathogen-associated molecular patterns
PCP	Poria cocos polysaccharide
PICRUSt2	Phylogenetic Investigation of Communities by Reconstruction of Unobserved States 2
PLs	Polysaccharide lyases
POP	Polygonatum odoratum polysaccharide
PPAR-γ	Peroxisome proliferator-activated receptor-γ
PULs	Polysaccharide utilization loci
Rha	Rhamnose
Rhap	Rhamnopyranose
RORγt	Retinoic acid receptor-related orphan receptor-γt
ROS	Reactive oxygen species
SCFAs	Short-chain fatty acids
SOD1/2	Superoxide dismutase 1/2
STAT3/5	Signal transducer and activator of transcription 3/5
TCM	Traditional Chinese medicine
Tfh13	T follicular helper 13 cells
TGF-β/TGF-β1	Transforming growth factor-β/transforming growth factor-β1
Th2/Th17	T helper 2/17 cells
THP	*Tetrastigma hemsleyanum* polysaccharide
TLR4/5	Toll-like receptor 4/5
TNF-α	Tumor necrosis factor-α
Treg	Regulatory T cells
Xyl	Xylose
ZO-1	Zonula occludens-1
α-SMA	α-Smooth muscle actin
